# Low-intensity pulsed ultrasound delays the progression of osteoarthritis by regulating the YAP–RIPK1–NF-κB axis and influencing autophagy

**DOI:** 10.1186/s12967-024-05086-x

**Published:** 2024-03-16

**Authors:** Chunran Pan, Fan Lu, Xiaoxia Hao, Xiaofeng Deng, Jiawei Liu, Kai Sun, Wenjie Hou, Xingru Shang, Ruimin Chi, Fengjing Guo, Tao Xu

**Affiliations:** 1 Department of Rehabilitation, Tongji Hospital, Tongji Medical College, Huazhong University of Science and Technology, 1095#, Jie-Fang Avenue, Qiaokou District, Wuhan, 430030 Hubei China; 2 Department of Orthopedics, Tongji Hospital, Tongji Medical College, Huazhong University of Science and Technology, 1095#, Jie-Fang Avenue, Qiaokou District, Wuhan, 430030 Hubei China

**Keywords:** Osteoarthritis, Chondrocyte, Autophagy, YAP, LIPUS

## Abstract

**Background:**

Osteoarthritis (OA) is a degenerative disease characterized by chronic inflammation of the joint. As the disease progresses, patients will gradually develop symptoms such as pain, physical limitations and even disability. The risk factors for OA include genetics, gender, trauma, obesity, and age. Unfortunately, due to limited understanding of its pathological mechanism, there are currently no effective drugs or treatments to suspend the progression of osteoarthritis. In recent years, some studies found that low-intensity pulsed ultrasound (LIPUS) may have a positive effect on osteoarthritis. Nonetheless, the exact mechanism by which LIPUS affects osteoarthritis remains unknown. It is valuable to explore the specific mechanism of LIPUS in the treatment of OA.

**Methods:**

In this study, we validated the potential therapeutic effect of LIPUS on osteoarthritis by regulating the YAP–RIPK1–NF-κB axis at both cellular and animal levels. To verify the effect of YAP on OA, the expression of YAP was knocked down or overexpressed by siRNA and plasmid in chondrocytes and adeno-associated virus was injected into the knee joint of rats. The effect of LIPUS was investigated in inflammation chondrocytes induced by IL-1β and in the post-traumatic OA model.

**Results:**

In this study, we observed that YAP plays an important role in the development of osteoarthritis and knocking down of YAP significantly inhibited the inflammation and alleviated cartilage degeneration. We also demonstrated that the expression of YAP was increased in osteoarthritis chondrocytes and YAP could interact with RIPK1, thereby regulating the NF-κB signal pathway and influencing inflammation. Moreover, we also discovered that LIPUS decreased the expression of YAP by restoring the impaired autophagy capacity and inhibiting the binding between YAP and RIPK1, thereby delaying the progression of osteoarthritis. Animal experiment showed that LIPUS could inhibit cartilage degeneration and alleviate the progression of OA.

**Conclusions:**

These results showed that LIPUS is effective in inhibiting inflammation and cartilage degeneration and alleviate the progression of OA. As a result, our results provide new insight of mechanism by which LIPUS delays the development of osteoarthritis, offering a novel therapeutic regimen for osteoarthritis.

**Supplementary Information:**

The online version contains supplementary material available at 10.1186/s12967-024-05086-x.

## Introduction

Osteoarthritis is one of the most common degenerative diseases characterized by chronic pain and destruction of articular cartilage [1]. As reported, OA primarily affects individuals over 50 years old and significantly impacts the quality of life for elderly patients to varying degrees [2]. Globally, more than 500 million people worldwide are influenced by OA, resulting in a substantial enormous economic burden on society and families [3]. At present, the treatment of OA mainly includes symptomatic treatment such as drugs and joint replacement surgery [4]. Unfortunately, the clinical diagnosis of OA is usually established later in the disease process, and drugs are often unable to provide more help [5]. To date, there is no effective methods to reverse the progression of OA [6]. Therefore, it is essential to explore novel therapeutic approaches for OA.

Autophagy, a normal cellular metabolism process, plays a vital role in energy regulation and the removal of damaged macromolecules and organelles [7, 8]. As reported, autophagy is the primary mechanism of articular chondrocytes to maintain normal function and cell survival. Dysregulation of autophagy can accelerate articular cartilage degeneration, while activation of autophagy delay the progression of OA [9, 10]. Meanwhile, we noticed a particular molecule. Yes-associated protein (YAP), a transcriptional coactivator, is involved in controlling cellular behavior and processes such as proliferation, differentiation, and migration [11, 12]. As reported, recent studies revealed the interaction between autophagy and the Hippo–YAP signaling pathways [13]. When autophagy is at a relatively low level, the activation of YAP is higher [14]. In addition, YAP plays a significant role in the progression of OA by responding to variable mechanical stress and is associated with the nuclear factor Kappa B (NF-κB) signaling pathway [15].

Ultrasound is an oscillating longitudinal pressure wave [16]. When cells are exposed to ultrasound, they experience mechanical stress. A recent study has shown that low-intensity pulsed ultrasound (LIPUS), a special type of ultrasound can promote cartilage repair after damage and slow down cartilage degeneration [17]. Furthermore, LIPUS converts acoustic signals into mechanical signals, which then trigger a series of cellular responses [18, 19]. For example, LIPUS has been reported to treat periodontitis and neurological diseases owing to its anti-inflammatory effect [20–23]. Notably, the effect of LIPUS in inhibiting apoptosis is remarkable, and LIPUS has shown positive effects on myocardial necrosis and nervous system diseases by inhibiting apoptosis of cardiomyocytes and neuronal cells [24, 25]. In addition, LIPUS also promote stem cell migration and proliferation and enhance cartilage repair by activating autophagy [26, 27]. What excited is that many studies have confirmed the effectiveness of nanomaterials in the treatment of diseases, and LIPUS has also shown new potential in the adjuvant treatment of nanomaterials in recent years, suggesting that LIPUS has great potential in the treatment of many diseases [28–31]. Furthermore, research has indicated that LIPUS can regulate the expression level of YAP under certain conditions [32, 33]. Nevertheless, the exact mechanism by which LIPUS acts in osteoarthritis through Hippo–YAP signaling pathway remains a puzzle. Therefore, it is of great significance to explore the specific mechanism of LIPUS on inflammatory chondrocytes for the clinical application of LIPUS in the treatment of OA.

In our study, we investigated the role of YAP and the function of LIPUS in inflammatory chondrocytes and post-traumatic OA rats. Additionally, we also examined whether LIPUS could inhibit the development of OA through regulating the YAP–RIPK1–NF-κB axis and autophagy.

## Materials and methods

### Reagents and materials

The list of regents was obtained commercially: Recombinant Rat interleukin 1 beta (IL-1β) (501-RL-010) was acquired from R&D Systems (Minneapolis, MN, USA). Rapamycin (Rapa) (S1039) was acquired from Selleck (Houston, USA) and diluted in DMSO. Antibodies against COX2 (12882; 1:1000), YAP (14074; 1:1000), P-P65 (3033; 1:1000), P-YAP (13008; 1:1000) and P65 (8242; 1:1000) were gained from Cell Signaling Technology Inc. (Beverly, USA). Antibodies against MMP13 (18165-1-AP; 1:1000), COL2A1 (28459-1-AP; 1:800), GAPDH (10494-1-AP; 1:5000), P-RIPK1 (66854-1-Ig; 1:2000) and β-actin (CL594-66009; 1:5000) were acquired from Proteintech Group (Wuhan, China). Antibodies against iNOS (A0312; 1:1000) and aggrecan (A11691; 1:1000) were acquired from Abclonal (Wuhan, China). Antibodies against P62 (GB11531-100; 1:1000) was acquired from Servicebio (Wuhan, China). Antibodies against MMP3 (BM4074; 1:500), FITC-conjugate goat anti-mouse and anti-rabbit secondary antibodies, type II collagenase and trypsin were purchased from Boster (Wuhan, China). IP lysis buffer (p0013) was provided by Beyotime (Shanghai, China).

### Chondrocytes isolation and culture

Isolated the primary chondrocytes from the knee joint of 5-day-old rats. All animals were acquired from the Laboratory Animal Center of Tongji Hospital and permitted by the institution of Animal Care and Use Committee of Tongji. The articular cartilages were excised under a sterile operating environment, and cartilage was sliced and then digested with 0.25% trypsin for 30 min at 37 °C. Subsequently, the cartilage was treated by type II collagenase for 5 h at 37 °C. The primary chondrocytes were harvested and cultured in TC flask T25 with dulbecco modified Eagle’s Culture Medium F12 (DMEM/F12) (Hyclone, Logan, UT, USA) consisting of 10% fetal bovine serum (Biological Industries, Israel). The medium was changed every 2 days until the chondrocytes were cultured to the second generation and used in the experiments.

### Small interfering RNA (siRNA), plasmids, and transfection

The negative control siRNA and siRNA of YAP were synthesized by RiboBio (Guangzhou, China). The three sequences are shown below: si-YAP #1: GCCATGAACCAGAGGATCA; si-YAP #2: GGCTGCGATTGAAACAGCA; si-YAP #3: CCAGACGCTGATGAACTCT. Chondrocytes were transfected with the Lipofectamine 3,000 transfection reagent according to the protocol (Thermo Fisher Scientific, Waltham, MA, USA) when the cell density reached 60–70%. The negative control plasmid and YAP overexpression plasmid was provided by GeneChem (Co. Ltd., Shanghai, China). Transfected the cells using Lipofectamine 3000 and P3000 when the fusion degree reached 60–70%, chondrocytes were washed and cultured in fresh medium after 24 h later.

### LIPUS stimulation

LIPUS exposure apparatus was purchased from ITO Corporation Ltd (Tokyo, Japan OSTEOTRONIV). Firstly, chondrocytes were transferred into a six-well plate and then exposed to LIPUS at an ultrasound frequency of 1.5 MHz and the pulse frequency of 1000 Hz, and the intensity was 30 mW/cm^2^, 45 mW/cm^2^ and 60 mW/cm^2^. The duration of the intervention was 10 min, 20 min and 30 min. Before intervention, a 2 mm thick coupler (Subijie, Chongqing, China) was applied between the bottom of the plate and the ultrasound probe. After exposed to LIPUS, chondrocytes were harvested immediately. In the meantime, the control group was put in the identical environment but except LIPUS stimulation.

### Western blot analysis

In briefly, disintegrated the treated chondrocytes with RIPA (Boster, China, AR0102) and sonicate the collections for 3 times on the ice. Then centrifuged the samples at 12,000×*g* and 4 °C for 30 min. Quantified supernatant by bicinchoninic acid method and next heated with suitable loading buffer at 95 °C for 10 min. Sodium dodecyl sulfate–polyacrylamide gel electrophoresis (SDS-PAGE) were filled with 25 mg of total protein per hole. The wet transmembrane was applied to transfer the protein to the polyvinylidene difluoride (PVDF) membrane at an electric current of 275 mA for 120 min. After membranes were blocked at room temperature for 1 h with tris-buffered saline (TBS) (Boster, AR0144) containing 5% skim milk powder and then incubated with the primary antibodies at 4 °C overnight. After that, membranes were incubated with corresponding secondary antibody at room temperature for 1 h. After wash the membranes, filmed the membranes with chemiluminescence (Boster, China). A Bio-Rad scanner was applied to detect the signal strength of the membranes.

### Quantitative RT-PCR

Using the centrifugal column-type ultrapure extract total RNA kit in line with the protocol (Omega Biotek, R6834-01) to extract the total RNA. And then synthesized the complementary DNA by a Hifair® III 1st Strand cDNA Synthesis SuperMix (Yeasen, 11141ES60). Then amplified the cDNA using the SYBR Green Master Mix (Yeasen, 11203ES03). The sequences of the primers were synthesized from Tsingke Biotechnology. GAPDH was served as a reference to normalize the relative expression. Primer sequence utilized in the study: Yap (F) 5′-TTTGCCATGAACCAGAGGAT-3′, (R) 5′-TATCTGCTGCTGCTGGTTTG-3′. Adamts5 (F) 5′-TCCTCTTGGTGGCTGACTCTTCC-3′, (R) 5′-TGGTTCTCGATGCTTGCATGACTG-3′. Mmp3 (F) 5′-CAGTCCTGCTGTGGCTGTGTAC-3′, (R) 5′-AACCTCCATGCCAGCATCTTCTTC-3′. MMP13 (F)5′-ACCATCCTGTGACTCTTGCG-3′, (R) 5′-TTCACCCACATCAGGCACTC-3′. Gapdh (F) 5′-ACAGCAACAGGGTGGTGGAC-3′, (R) 5′-TTTGAGGGTACAGCGAACTT-3′. Each test was repeated in triplicate.

### Co-immunoprecipitation (Co-IP) assay

The primary chondrocytes were cultured and treated with IL-1β for 24 h in 10 cm dishes. Afterwards, cells were lysed in 0.5 mL of IP lysis buffer with appropriate protease inhibitors. Centrifugate the collections at 4 °C for 15 min after lysed it in the ice for 10 min. Then, added Protein A+G magnetic beads into the cell lysates and shaking for 1 h to remove non-specific binding. After that, washed the magnetic beads for four times with lysis buffer. Anti-YAP antibody was put into the mixture and shaking overnight at 4 °C in order to form immune complexes with the protein of YAP. Finally, some lysis buffer and suitable 5X loading buffer were added. After boiling the mixture for 10 min at 97 °C, the supernatant was converged for western blot analysis.

### The detection of autophagic flux

The mRFP-GFP-LC3 adenovirus was gained from Hanbio Biotechnology (HB-AP210 0001). Firstly, inoculated the chondrocytes into 35 mm glass bottom confocal culture dish. Transfect the cells with mRFP-GFP-LC3 adenovirus for 6 h followed by medium change. After cultured with fresh medium for 24 h, the chondrocytes were treated with IL-1β and then fixed with 4% paraformaldehyde (PFA) (Boster, China). Subsequently, captured the images by FV3000 confocal microscope (Olympus, Japan). In the pictures, the red highlights represent the autophagolysosomes and the yellow highlights (overlapped by green and red fluorescence) represent the autophagosomes.

### Immunofluorescence (IF) assay and confocal microscopy

Chondrocytes were seeded into 35 mm glass bottom confocal culture dish and treated as mentioned in this article. Subsequently, fixed the samples by 4% PFA for 10 min and used with 0.5% Triton X-100 (Boster, China) to permeabilization the cell membrane. After that, blocked the chondrocytes with goat serum for 1 h. Next, incubated chondrocytes with primary antibodies of MMP13 (1:200), COL2A1 (1:200), YAP (1:200), P65 (1:500), P-RIPK1 (1:200) and P62 (1:500), as well as the corresponding second antibodies. After incubation, stained the nucleus with DAPI for 5 min. Finally, the images were captured by a laser scanning confocal microscope (FV3000, Olympus, Japan). The mean fluorescence intensity was calculated by ImageJ 1.46 r software (Java 1.6.0-20; Media Cybernetics, Rockville, MD, USA).

### Animals

Four-weeks-old male Sprague–Dawley (SD) rats were applied to animal experimental and acquired from the Laboratory Animal Center of Tongji Hospital. All animal experiments were permitted by the institution of Animal Care and Use Committee of Tongji (TJH-202208009, Wuhan, China) and conformed to the stipulations in the World Medical Association Helsinki Declaration. All rats were bred in pathogen-free cages, allowed to move freely and provided autoclaved food and water. Besides, rats were housed in a 12-h dark–light cycle at controlled temperature of (22 ± 1) °C and humidity (55 ± 10%). After 1 week of acclimatization, forty-eight rats were randomly divided into six groups: Sham group, DMM group, DMM + KD-NC group, DMM + KD-YAP group, DMM + OE-NC group, DMM + OE-YAP group, with 8 rats in each group. Destabilization of medial meniscus (DMM) surgery was managed on the right knee joint to established the model of post-traumatic OA. The Sham group underwent sham operation of the medial joint capsule incision as a control. Four weeks before surgery, rats except Sham group and DMM group were injected with adeno-associated virus (AAV) (1 × 10^11^ v.g) into the joint cavity. In addition, to evaluate the effect of LIPUS on the expression level of YAP and the development of OA. Forty-eight rats were indiscriminately separated into 6 groups averagely. Sham group, Sham + LIPUS group, DMM group, DMM + LIPUS group, DMM + OE-YAP group and DMM + OE-YAP + LIPUS group were established as described previously. One week after DMM surgery, the rats in Sham + LIPUS group, DMM + LIPUS group and DMM + OE-YAP + LIPUS group were treated with LIPUS (30 mW/cm^2^, 1.5 MHz, 1000 Hz, 20 min/day, 5 days/week, 6 weeks). At the same time, recorded the body weight of each rat once a week. At the end of the intervention, the operated joints were taken for micro-CT, histological staining and immunohistochemistry.

### Pain assessment

Nociception to heat was tested using the Hargreaves plantar test (IITC Life Science, CA, USA). The rats were put on plastic box placed on a glass panel, focus radiation heat sources below the damage site at 20-s intervals to prevent potential thermal damage. The incubation period was averaged over the three tests, 5 min apart. The withdrawal thresholds for mechanical stimulation were measured by an electronically controlled von Frey filament system (IITC Life Science). The device consists of a recording device, a handle and a rigid plas tic disposable tip with a diameter of 0.8 mm. A positive response was manifested as a rapid withdrawal or licking of the hind paw of the rat, followed by the recording of chrono intensity values. Experimental measurements were performed every 2 weeks. All the rats were stimulated after 20–30 min of acclimatization in the plastic box.

### Enzyme-linked immunosorbent assay (ELISA)

Firstly, collected and stored the serum of each rat. Then, IL-1β (MM-0047R1, MEIMIAN, China) and tumor necrosis factor α (TNF-α) (RA20035, Bioswamp) level from the serum were detected according to the ELISA kit instructions.

### Micro-computed tomography (micro-CT) analysis

In brief, collect the right knee joint and fix it with 4% paraformaldehyde for 3 days. Then, scan and detect the knee by micro-CT with a micro-CT 50 scanner (Scanco Medical, Switzerland). The scanning parameter was 70 kv and 230 ms with the thickness of 19 μm. The reconstructed three-dimensional (3D) images were obtained by Scanco Medical software. The abrasion of the tibial plateau was evaluated.

### Histological staining and immunohistochemistry

Firstly, fixed the tissue samples with 4% PFA for 3 days, decalcified it for 60 days with 10% EDTA solution. Subsequently, cut it into 4-μm thick sagittal sections after embedded the samples in paraffin. Then, staining the specimens with Safranin O/Fast green and Hematoxylin–eosin (H&E). The Osteoarthritis Research Society International (OARSI) histopathology scoring system was used to assess the cartilage degeneration. After deparaffinized and rehydrated the samples, using the BSA involved in 0.1% Triton X-100 to block the samples for 1 h. Next, incubate the samples with anti-COL2A1, anti-P-P65, anti-P-YAP, anti-P62, anti-P-RIPK1, anti-MMP13 or anti-YAP antibodies, then incubate the corresponding secondary antibody and colored with DAB. Lastly, apply the hematoxylin to counterstained the specimens. Catch the images with a general microscope (BX53, Olympus Corporation, Japan). Each section was assessed by two blinded researchers.

### Statistical analysis

Dates were analyzed by GraphPad Prism 8.0.2 program (GraphPad Software, San Diego, CA, USA) and presented as mean ± SD. Experiments were repeated based on cells isolated from at least three animals and independently repeated three times. One-way analysis of variance (ANOVA) was applied for multiple experimental groups. P < 0.05 was defined as significant, * means P < 0.05, ** means P < 0.01, *** means P < 0.001 while NS represents not significant.

## Result

### OA was characterized by altered expression of YAP

**Fig. 1:**
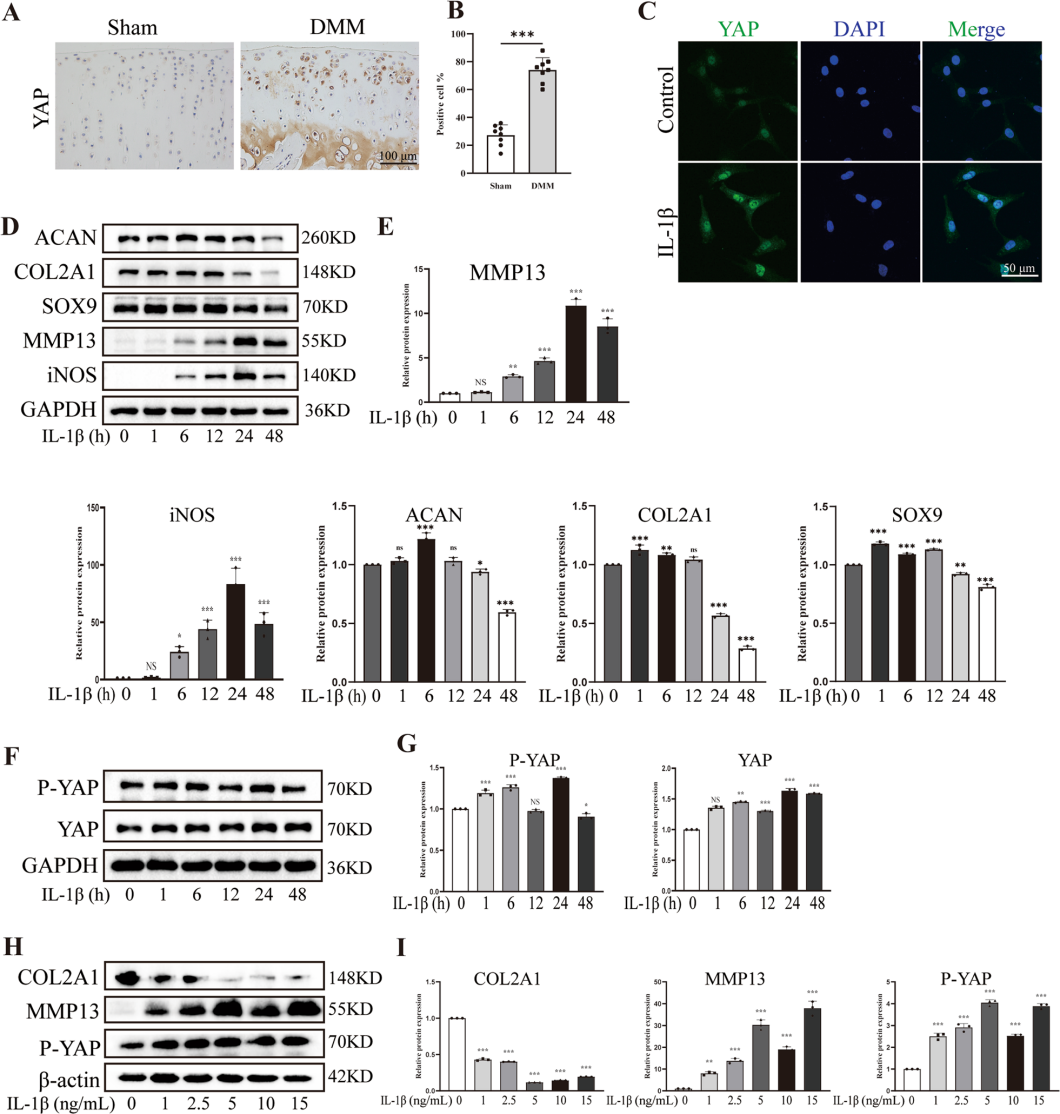
The expression of YAP in OA. **A**, **B** Immunohistochemistry images and quantitative analysis of YAP-positive cells in knee joint cartilage of rats (Sham group: n = 8; DMM group: n = 8, scale bar: 100 μm). **C** Immunofluorescence image of fluorescence intensity to show the relative expression of YAP in chondrocytes treated with IL-1β for 24 h (scale bar: 50 μm). **D**, **E** Western blot of the indicated protein level to demonstrate that IL-1β treat the chondrocytes for 0, 1, 6, 12, 24 and 48 h could cause inflammation of chondrocytes. **F**, **G** Western blot and quantitative analysis of P-YAP and YAP band density. **H**, **I** Western blot and quantitative analysis of COL2A1, MMP13 and P-YAP band density. Data are shown as mean ± SD. *P < 0.05, **P < 0.01, ***P < 0.001

To investigate the relationship between YAP and the development of OA, we performed DMM surgery on SD rats to establish a post-traumatic OA model. Immunohistochemistry results revealed a significant increase in YAP-positive chondrocytes in the cartilage of DMM surgery rats (Fig. 1A, B). Furthermore, we isolated primary rat chondrocytes and evaluated the expression level of YAP in vitro. Notably, Pro-inflammatory factors are known to play a crucial role in the occurrence and progression of OA [34]. To verify the expression of YAP in inflammatory chondrocytes, we applied IL-1β to establish the inflammatory-degenerative cell model. Consistent with above, the IF staining confirmed a distinct increase in YAP expression in the chondrocytes treated with IL-1β (Fig. 1C). Moreover, western blot results revealed that as the duration of IL-1β intervention increased, the expression of anabolic related factors, including collagen type II α 1 chain (COL2A1), SOX9 and aggrecan (ACAN) decreased. On the other hand, the expression of catabolism protein such as matrix metallopeptidase 13 (MMP13) and inflammatory markers including inducible nitric oxide synthase (iNOS) exhibited an opposite trend (Fig. 1D, E). In addition, the expression of P-YAP and YAP also increased especially at the duration of 24 h (Fig. 1F, G). Furthermore, as the concentration of IL-1β increased, the expression of anabolic related factors such as COL2A1 decreased, whereas the expression of catabolism protein such as MMP13 gradually increased. Notably, when the concentration of IL-1β was 5 ng/mL, the expression of P-YAP was higher compared to other groups, meaning that the concentration of 5 ng/mL and the duration of 24 h of IL-1β treatment establishes inflammatory state in chondrocytes successfully (Fig. 1H, I). Collectively, these results indicated the expression of YAP is increased in the articular cartilage of post-traumatic OA rats and is further enhanced in response to IL-1β intervention, which demonstrated a positive correlation between YAP and the progression of OA.

### Autophagy mediates protective role in IL-1β-induced detrimental events

**Fig. 2:**
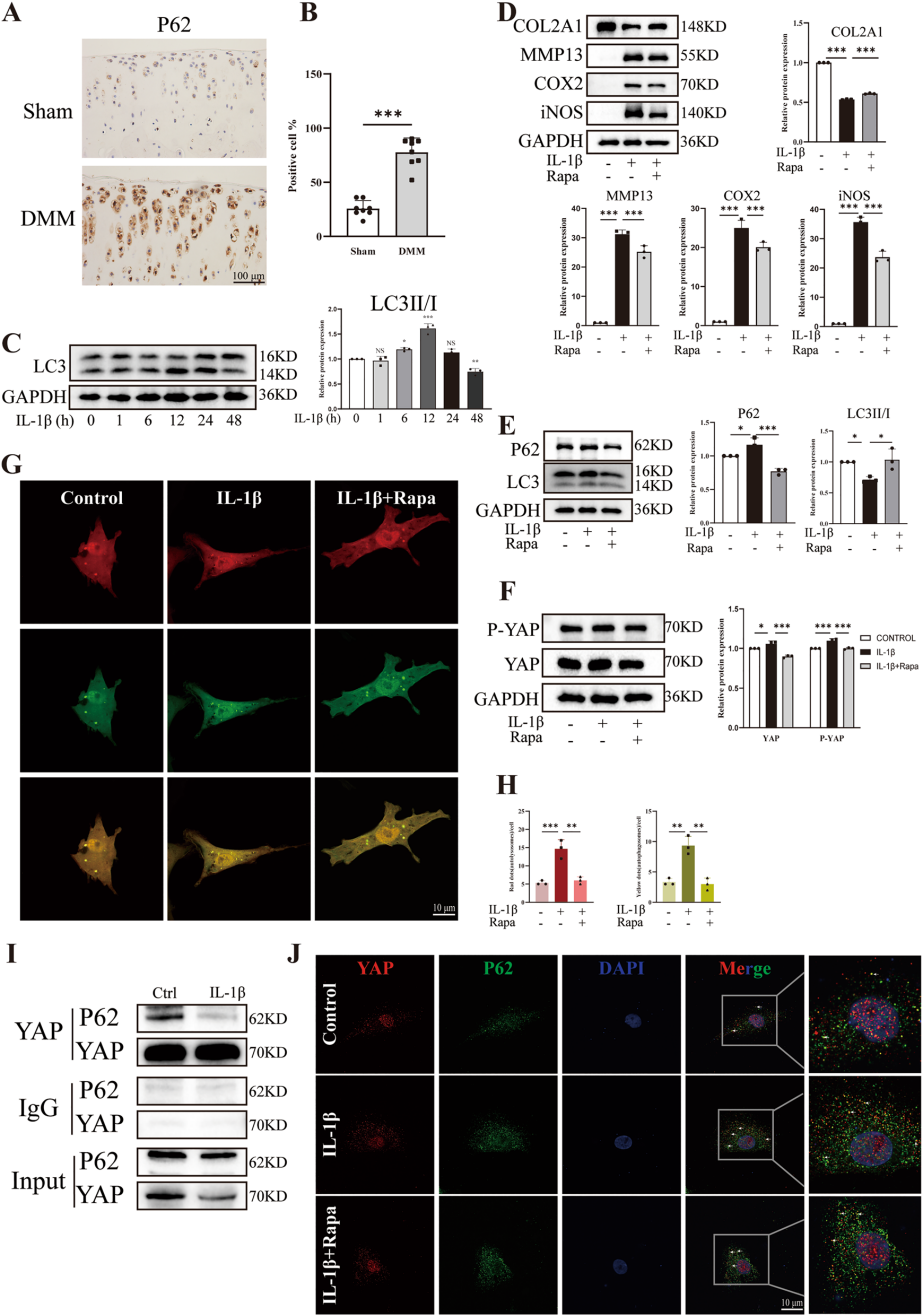
Autophagy is associated with OA. **A**, **B** Immunohistochemistry images and quantitative analysis of P62-positive cells in knee joint cartilage of rats (sham group: n = 8; DMM group: n = 8, scale bar: 100 μm). **C** Western blot and quantitative analysis of LC3 expression level after IL-1β treatment the chondrocytes for 0, 1, 6, 12, 24 and 48 h. **D** Western blot and quantitative analysis of COL2A1, MMP13, COX2 and iNOS expression level. **E** Western blot and quantitative analysis of P62 and LC3 expression level. **F** Western blot and quantitative analysis of P-YAP and YAP expression level. **G**, **H** Chondrocytes were transfected with mRFP-GFP-LC3 adenovirus following IL-1β treatment for 24 h. The representative images of fluorescence and quantitative analysis of red dots (autolysosomes) and yellow dots (autophagosomes) were shown (scale bar: 10 μm). **I** Co-IP experiment of the binding between YAP and P62 was shown. **J** YAP and P62 colocalization in chondrocytes were detected by IF (scale bar: 10 μm). Data are shown as mean ± SD. *P < 0.05, **P < 0.01, ***P < 0.001

Previous studies have shown that autophagy is impaired during the development of OA. Besides, in our study, we observed an increase in the expression of YAP and P-YAP in inflammatory chondrocytes. Based on these findings, we hypothesize that the accumulation of YAP in chondrocytes is due to the impaired autophagy. To investigate the relationship between YAP and autophagy, we examined the expression of P62 (an autophagy-related gene) in cartilages of rats underwent DMM surgery. Immunohistochemistry results showed higher expression of P62 in the cartilage of DMM surgery rats compared to Sham rats (Fig. 2A, B). Besides, western blot analysis also revealed an increase in the rate of microtubule-associated protein 1 light chain 3β (LC3), a marker of autophagy, as the duration of IL-1β intervention increased (Fig. 2C). To further explore the connection between autophagy impairment, inflammation and YAP accumulation, we treated chondrocytes with Rapamycin, which is the activator of autophagy. The western blot results revealed that IL-1β not only promoted the degradation of cartilage extracellular matrix (ECM) and the inflammatory process (Fig. 2D), but also upregulated the expression of P62 and downregulated the ratio of LC3II/I as well as accelerated the accumulation of YAP (Fig. 2E, F). These results indicated that the autophagy of chondrocytes was impaired after IL-1β intervention, which resulted in the decrease of the ability of cells to digest YAP and the increase of intracellular accumulation of YAP. Furthermore, to investigate the occurrence of autophagic influx, we used tandem fluorescence-tagged LC3 adenovirus (RFP-GFP-LC3) to infect chondrocytes and found an increase in yellow puncta in chondrocytes treated with IL-1β compared to the control group (Fig. 2G, H). These findings demonstrated that IL-1β impairs the macroautophagy/autophagic flux and promotes the transformation of autophagosomes into autolysosomes. Interestingly, inflammatory chondrocytes treated with Rapamycin reversed these negative effects. In addition, to further confirm the direct association between YAP and autophagy and its influence on inflammatory chondrocytes, we conducted a Co-IP assay. The data revealed that YAP could bind to P62 (Fig. 2I). Consistently, the IF colocalization assay demonstrated the increased colocalization of YAP with P62 in inflammatory chondrocytes, which was reduced by Rapamycin (Fig. 2J). In summary, the impaired autophagy capacity in inflammatory chondrocytes leads to the accumulation of YAP and inflammatory response in chondrocytes.

### YAP contributed OA-like phenotype of chondrocytes

**Fig. 3:**
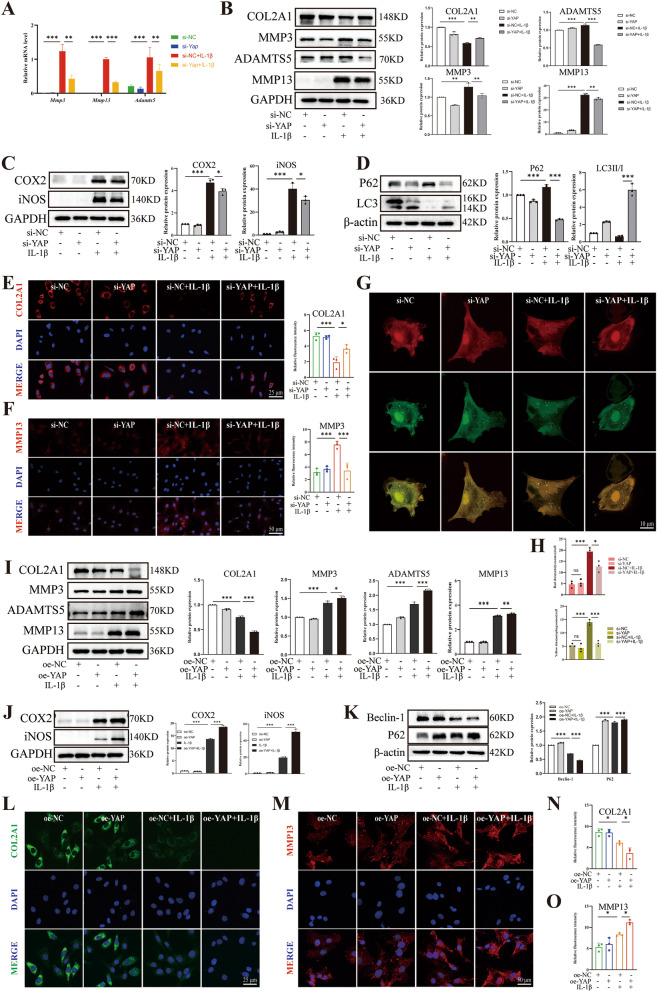
YAP exacerbated chondrocyte damage induced by IL-1β. Chondrocytes transfected with si-NC or YAP siRNA following IL-1β induction for 24 h. **A** qPCR of MMP3, MMP13 and ADAMTS5 relative expression level. **B** Western blot and quantitative analysis of COL2A1, MMP3, ADAMTS5 and MMP13 expression level. **C**, **D** Western blot and quantitative analysis of COX2, iNOS, P62 and LC3 expression level. **E**, **F** Immunofluorescence staining and fluorescence intensity analysis of COL2A1 and MMP13 relative expression level. **G**, **H** Chondrocytes were transfected with si-NC or si-YAP and then transfected with mRFP-GFP-LC3 adenovirus following IL-1β treatment for 24 h. The representative images of fluorescence and quantitative analysis of red dots and yellow dots were shown (scale bar: 10 μm). Subsequently, Chondrocytes transfected with oe-NC or oe-YAP following IL-1β induction for 24 h. **I** Western blot and quantitative analysis of COL2A1, MMP3, ADAMTS5 and MMP13 expression level. **J**, **K** Western blot and quantitative analysis of COX2, iNOS, Beclin-1 and P62 expression level. **L**–**O** Immunofluorescence staining and fluorescence intensity analysis of COL2A1 and MMP13 relative expression level. Data are shown as mean ± SD. *P < 0.05, **P < 0.01, ***P < 0.001

To further validate the role of YAP in the development of OA, we investigated the effects of knocking down and overexpressing of YAP on inflammatory chondrocytes. Firstly, we confirmed the efficiency of YAP knocking down using siRNA by qPCR (Additional file 1: Fig. S1A). Chondrocytes play a crucial role in maintaining ECM homeostasis in the bone and joint. Therefore, we examined changes in ECM metabolism after changing the expression of YAP. The results showed that chondrocytes exhibited ECM degradation after treated with IL-1β. However, when chondrocytes were treated with IL-1β and si-YAP, the ECM degradation was significantly reversed and inflammation was attenuated. First, qPCR analysis revealed that the mRNA levels of *Mmp3*, *Mmp13* and *Adamts5* were decreased compared to the IL-1β group (Fig. 3A), indicating that YAP deficiency significantly downregulated the expression of catabolism related genes in inflammatory chondrocytes at the gene level. Furthermore, western blot results reveled that when chondrocytes were treated with IL-1β and si-YAP, the expression of anabolic marker such as COL2A1 was increased, while the expression of catabolic proteins such as metalloproteinase 3 (MMP3), MMP13 and a disintegrin and metalloproteinase with thrombospondin motifs 5 (ADAMTS5) were decreased (Fig. 3B), suggesting that YAP deficiency restored ECM homeostasis in inflammatory chondrocytes. Additionally, YAP deficiency also showed a reduction in IL-1β-induced inflammatory response, as evidenced by the decreased levels of cyclooxygenase-2 (COX2) and iNOS (Fig. 3C). Notably, YAP deficiency was able to improve the impairment of autophagy induced by IL-1β, as showed by a decrease in P62 expression and an increase in the ratio of LC3II/I expression (Fig. 3D), indicating that YAP deficiency markedly inhibited the progression of inflammation and impaired autophagy in inflammatory chondrocytes. A resembling trend was observed in the immunofluorescence assay, revealing the negative effect of YAP in chondrocytes. when chondrocytes were treated with IL-1β and si-YAP, the fluorescence intensity of MMP13 was reduced compared to chondrocytes treated with IL-1β only, whereas COL2A1 shown the opposite trend (Fig. 3E, F). Moreover, in order to further observe the changes of autophagic flow in inflammatory chondrocytes tread with si-YAP, we infected chondrocytes with mRFP-GFP-LC3 adenovirus and found that IL-1β increased the number of yellow puncta, whereas chondrocytes treated with IL-1β and si-YAP resulted in fewer yellow puncta (Fig. 3G, H), suggesting YAP deficiency incompletely blocked the transformation from autophagosome to autophagolysosomes and restricted the macroautophagy/autophagy flux. Subsequently, we investigated the relevance of YAP overexpression in chondrocytes to further validate the above data. Western blot results suggested a significant increase in YAP expression in chondrocytes with YAP-plasmid transfection (Additional file 1: Fig. S1B, C). Contrary to the above data, in comparison to that treated with IL-1β only, YAP overexpression significantly reduced the expression of COL2A1 but increased the expression of MMP13, MMP3 and ADAMTS5 (Fig. 3I), as well as COX2 and iNOS (Fig. 3J), indicating that YAP overexpression considerably accelerated the imbalance of ECM homeostasis and inflammatory progression. Simultaneously, YAP overexpression further inhibited autophagy capacity, as shown by higher expression of P62 and downregulated levels of Beclin-1 (Fig. 3K), which revealed that YAP overexpression significantly aggravated the impairment of autophagy. Furthermore, the immunofluorescence assay also showed a similar trend, as showed by YAP overexpression increased the fluorescence intensity of MMP13, whereas COL2A1 shown the opposite trend (Fig. 2L–O). In conclusion, these results suggested that it was YAP contributes to the OA-like phenotype of chondrocytes and inhibits the capacity of autophagy.

### YAP overexpression exacerbated cartilage degeneration in post-traumatic OA rat

**Fig. 4:**
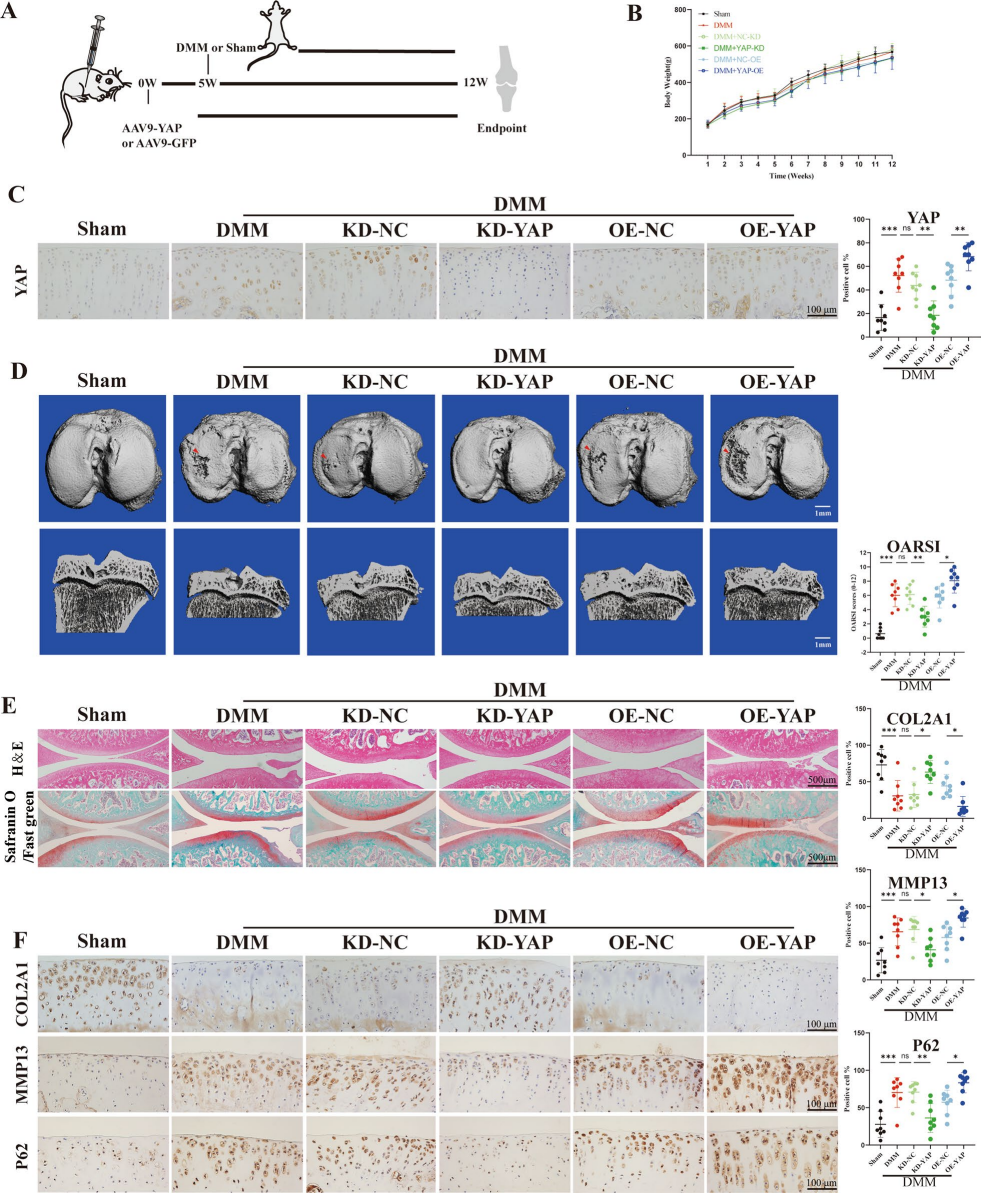
YAP overexpression exaggerates cartilage degeneration in post-traumatic OA rats. **A** Schematic diagram of animal experiment. **B** The body weight of rats. **C** IHC staining to show the YAP-positive cell in cartilage (scale bar: 100 μm). **D** The CT three-dimensional reconstruction images of rats (scale bar: 1 mm). **E** H&E staining and Safranin O/Fast green of the six groups (scale bar: 500 μm). **F**, **G** Immunohistochemistry staining to show the COL2A1-positive cell, MMP13-positive cell and P62-positive cell in cartilage of the six groups (scale bar: 100 μm). Data are shown as mean ± SD. *P < 0.05, **P < 0.01, *** P < 0.001

To further investigate the relationship between YAP and OA development, we established an experimental OA model. YAP knockdown or overexpression was achieved by injecting adeno-associated virus into the articular cavity of rats. Four weeks after the initial injection of AAV, rats underwent either a Sham surgery or a DMM surgery (Fig. 4A). There were no significant differences in body weight among the different groups (Fig. 4B). The results revealed that AAV injection significantly increased or decreased the expression of YAP in cartilage compared to the control group (Fig. 4C). Furthermore, compared to the Sham group, obvious subchondral bone damage was observed in the DMM group. Notably, the subchondral bone damage in rats treated with AAV9-YAP was more severe than that of the DMM group. Conversely, YAP knocking down alleviated these damage (Fig. 4D). Consistent with the above, we observed the identical trend in histological staining results. As expected, H&E and Safranin O/Fast green staining indicated significant cartilage degeneration in DMM group, as evidenced by the loss of proteoglycan and higher OARSI scores compared to the Sham group. Notably, YAP overexpression was more likely to promote DMM-induced degeneration, as indicated by higher OARSI scores compared to the OE-NC group. In contrast, YAP knocking down showed milder cartilage degeneration (Fig. 4E). Moreover, we further determined whether YAP overexpression affects the ECM metabolic markers and autophagy capacity. The results showed that the expression of MMP13 and P62 in the cartilage of the DMM group was significantly increased, which was higher after YAP overexpression, whereas COL2A1 shown the opposite trend. However, YAP knocking down inhibited the expression of MMP13 and P62 but promote the expression of COL2A1 (Fig. 4F). Collectively, these results suggest that YAP overexpression exacerbates the cartilage degeneration in post-traumatic OA rat.

### YAP interacts with RIPK1 to promote chondrocyte damage via the NF-κB signaling pathway

**Fig. 5:**
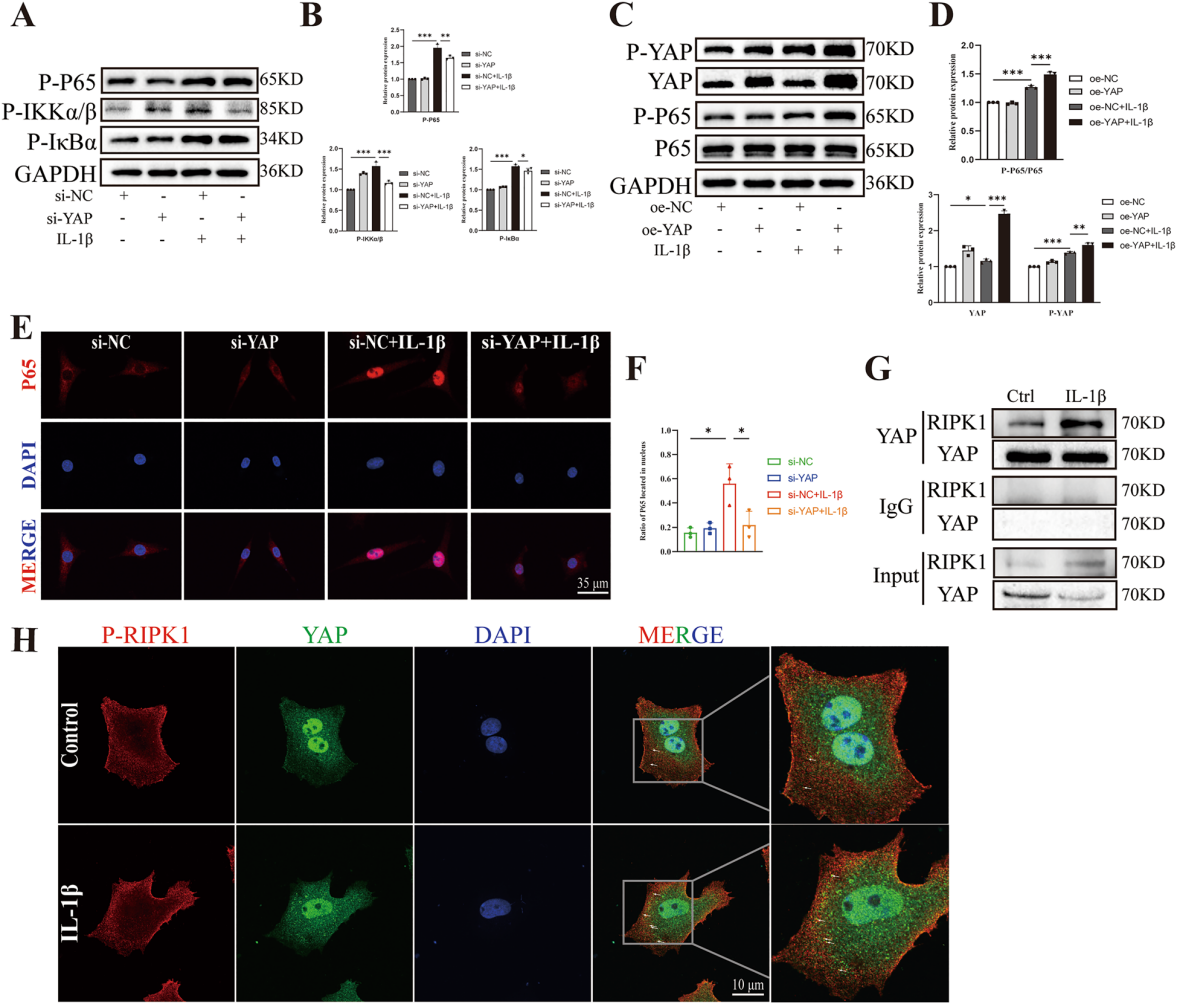
YAP regulates NF-κB signaling pathway. **A**, **B** Western blot and quantitative analysis of P-P65, P-IKKα/β and P-IκB relative protein expression with si-NC or si-YAP following IL-1β induction. **C**, **D** Western blot and quantitative analysis of P-YAP, YAP, P-P65 and P65 relative protein expression with oe-NC or oe-YAP following IL-1β induction. **E**, **F** Images of IF staining and statistical analysis for P65 relative expression and the distribution in chondrocytes transfected with si-NC or si-YAP following IL-1β induction for 15 min (scale bar: 35 μm). **G** Co-IP experiment of the binding between YAP and RIPK1 was shown. **H** YAP and P-RIPK1 colocalization in chondrocytes were detected by IF (scale bar: 10 μm). Data are shown as mean ± SD. *P < 0.05, **P < 0.01, ***P < 0.001

In addition to examine the OA-like phenotype indicators, we also investigated the NF-κB signaling pathway. The western blot results showed that knockdown of YAP reduced the phosphorylation of P65, IKKα/β and IκBα compared to the IL-1β group (Fig. 5A, B). Conversely, YAP overexpression exacerbated the phosphorylation of YAP and P65, indicating that YAP indeed affected the NF-κB signaling pathway (Fig. 5C, D). Furthermore, the immunofluorescence results confirmed that knocking down of YAP reduced the translocation of P65 to the nucleus (Fig. 5E, F). P65 is a nuclear factor that regulates transcription by translocating to the nucleus and phosphorylating to stimulate the expression of downstream genes [35], which is consistent with our finding that knocking down of YAP reduced the translocation of P65 to the nucleus, thereby decreasing the inflammation level of chondrocytes. Furthermore, we conducted a Co-IP experiment and discovered that YAP could bind to RIPK1, and IL-1β treatment increased the binding (Fig. 5G). The IF colocalization images also demonstrated that YAP could bind to RIPK1 and IL-1β treatment caused more binding (Fig. 5H). These results indicate that YAP can bind to and interact with RIPK1, which is an upstream molecule of the NF-κB signaling pathway, thereby regulating the NF-κB signaling pathway to aggravate chondrocyte degeneration and inflammation.

### LIPUS alleviates the development of OA

**Fig. 6:**
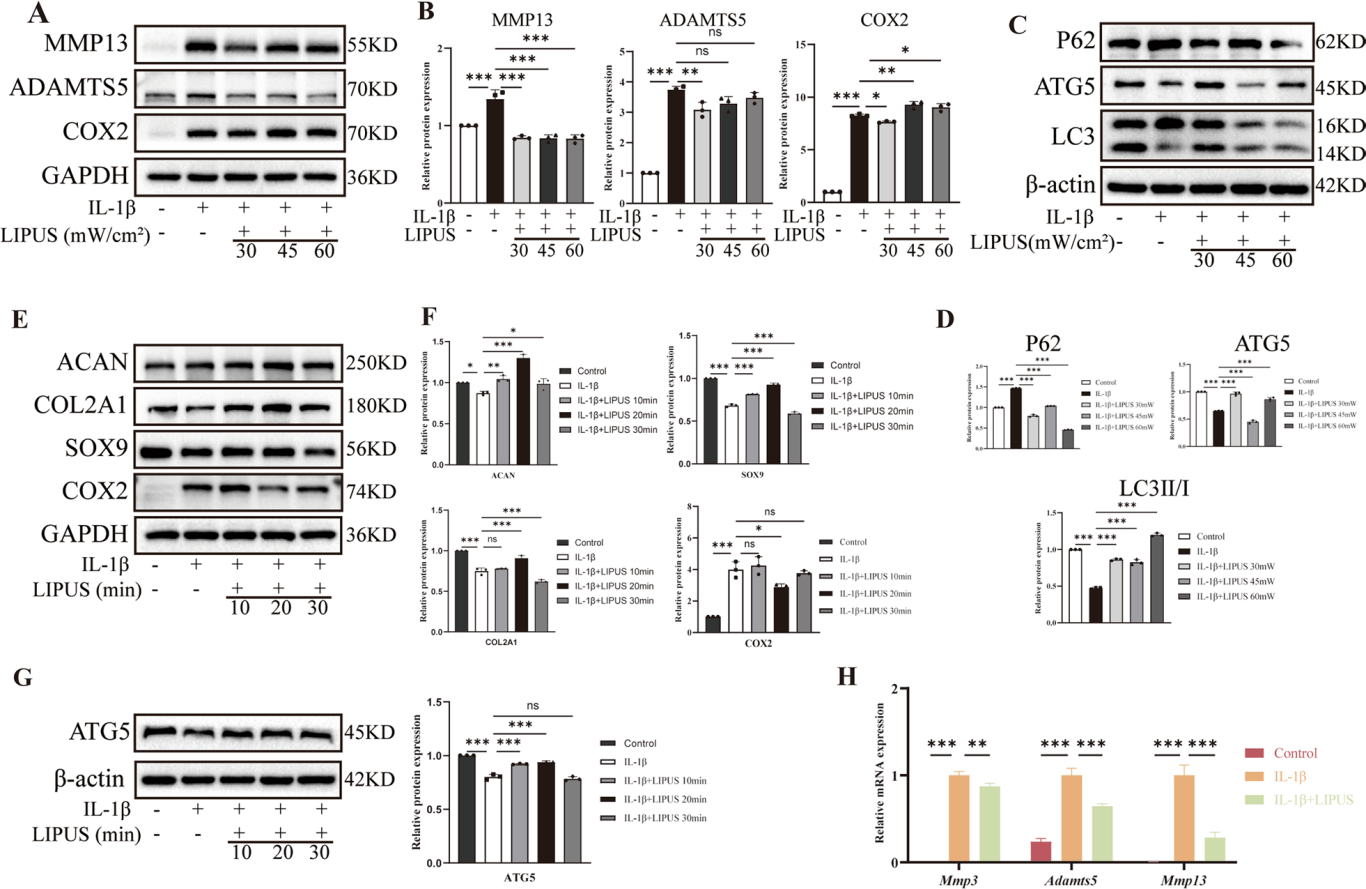
LIPUS inhibits the progression of OA. **A**, **B** Western blot and quantitative analysis of MMP13, ADAMTS5 and COX2 expression level with LIPUS application for 20 min. **C**, **D** Western blot and quantitative analysis of P62, ATG5 and LC3 expression level with LIPUS application for 20 min. **E**, **F** Western blot and quantitative analysis of ACAN, COL2A1, SOX9 and COX2 expression level with 30 mW/cm^2^ LIPUS application. **G** Western blot and quantitative analysis of ATG5 expression level with 30 mW/cm^2^ LIPUS application. **H** qPCR of MMP3, ADAMTS5 and MMP13 relative expression level. Data are shown as mean ± SD. *P < 0.05, **P < 0.01, ***P < 0.001

To investigate the effect of LIPUS on inflammatory chondrocytes, we conducted a screening process to determine the optimal intervention conditions in terms of intensity and duration. Firstly, chondrocytes were exposed to intensities of 30 mW/cm^2^, 45 mW/cm^2^, and 60 mW/cm^2^ for a duration of 20 min. The results indicated that the intensity of 30 mW/cm^2^ was most effective in reducing the expression of catabolism proteins (MMP13 and ADAMTS5) and inflammatory protein (COX2) (Fig. 6A, B). Furthermore, the intensity of 30mW/cm^2^ also induced autophagy in inflammatory chondrocytes, as evidenced by decreased expression of P62 and increased expression of autophagy-related gene 5 (ATG5) and LC3II/I (Fig. 6C, D). These results suggested that the intensity of 30 mW/cm^2^ significantly restored ECM homeostasis and inhibited the progression of inflammation and impaired autophagy in inflammatory chondrocytes. Subsequently, chondrocytes were treated with LIPUS for 10 min, 20 min and 30 min at an intensity of 30 mW/cm^2^ and we found that 20 min of treatment was most effective in upregulating the expression of anabolism proteins (ACAN, COL2A1 and SOX9), indicating the chondrogenic effect of LIPUS on chondrocytes (Fig. 6E, F). Additionally, 20 min of treatment also increased the expression of ATG5 (Fig. 6G). These results suggested that 20 min of treatment considerably restored ECM homeostasis and inhibited the impaired autophagy in inflammatory chondrocytes. Therefore, we selected the intensity of 30 mW/cm^2^ and the duration of 20 min as the intervention condition for subsequent experiments. In addition, the results of qPCR confirmed the positive effect of LIPUS in inflammatory chondrocytes (Fig. 6H).

**Fig. 7:**
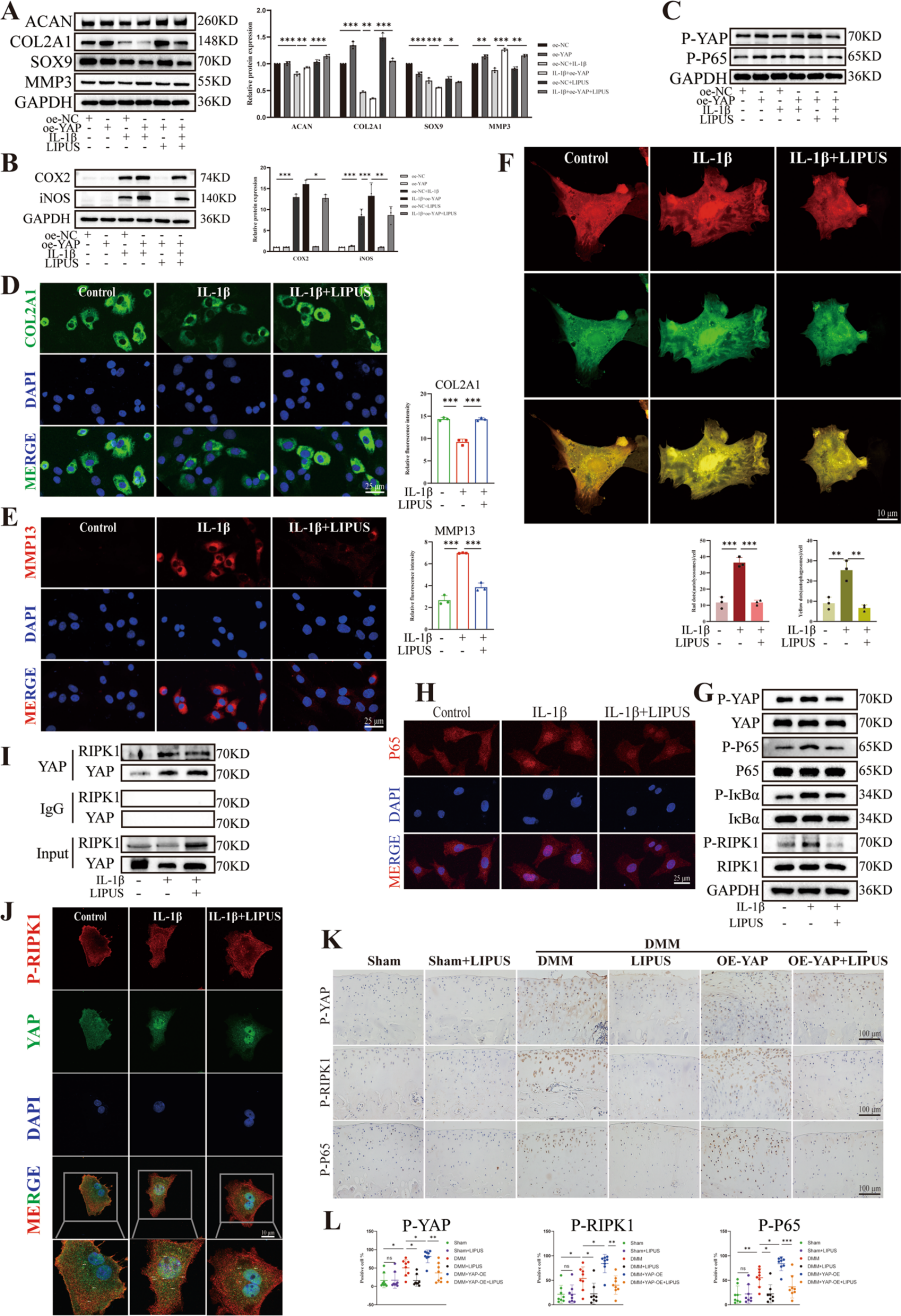
LIPUS inhibits the progression of OA via YAP/RIPK1 axis. **A** Western blot and quantitative analysis of ACAN COL2A1, SOX9 and MMP3 expression level. **B** Western blot and quantitative analysis of COX2 and iNOS expression level. **C** Western blot of P-YAP and P-P65 expression level. **D**, **E** Immunofluorescence staining and fluorescence intensity analysis of COL2A1 and MMP13 relative expression level. **F** Chondrocytes were transfected with mRFP-GFP-LC3 adenovirus following IL-1β treatment for 24 h. The representative images of fluorescence and quantitative analysis of red dots and yellow dots were revealed (scale bar: 10 μm). **G** Western blot of P-YAP, YAP, P-P65, P65, P-IκB, IκB, P-RIPK1 and RIPK1 relative protein expression. **H** Images of IF staining for P65 relative expression and the distribution in chondrocytes with IL-1β induction for 15 min (scale bar: 25 μm). **I** Co-IP experiment of the binding between YAP and RIPK1 after LIPUS intervention. **J** YAP and RIPK1 colocalization in chondrocytes were detected by IF after LIPUS intervention (scale bar: 10 μm). **K**, **L** Immunohistochemistry staining to show the P-YAP-positive cell, P-RIPK1-positive cell and P-P65-positive cell in cartilage of the six groups (scale bar: 100 μm). Data are shown as mean ± SD. *P < 0.05, **P < 0.01, ***P < 0.001

Subsequently, LIPUS was applied to further elucidate the therapeutic effect of on OA through regulating YAP. Westen blot results suggested that compared to chondrocytes treated with oe-YAP or IL-1β alone, treatment combination with oe-YAP and IL-1β further reduced the expression of COL2A1, ACAN and SOX9 but increased the expression of MMP3, COX2 and iNOS. However, LIPUS was able to reverse these adverse outcomes (Fig. 7A, B). These results indicated that LIPUS considerably reversed the imbalance of ECM homeostasis and inflammatory progression that accelerated by YAP overexpression. Moreover, Western blot results also showed that YAP overexpression increase the phosphorylation of YAP and P65 compared to the IL-1β group, while LIPUS demonstrated therapeutic effect (Fig. 7C), indicating that LIPUS regulated the phosphorylation of YAP and P65. Subsequently, we performed an IF assay, which indicated that the proliferation in COL2A1 fluorescence intensity increased after LIPUS intervention compared to the IL-1β group, whereas MMP13 showed the opposite trend (Fig. 7D, E). After that, we examined the effect of LIPUS on chondrocyte autophagic influx. The IF results revealed that LIPUS could partially restore the impaired autophagy capacity caused by IL-1β (Fig. 7F). In addition to the synthetic, catabolic and inflammation markers, we also examined NF-κB pathway related indicators and results shown that IL-1β administration increased the expression of P-YAP, P-P65, P-IκBα and P-RIPK1, while the application of LIPUS noticeably reduced the phosphorylation of YAP, P65, IκBα and RIPK1 (Fig. 7G). Notably, compared to the control group, the results of IF showed that treating the chondrocytes with IL-1β resulted in more transfer of P65 into the nucleus, which was decreased after exposed to LIPUS (Fig. 7H), indicating that LIPUS inhibited the nuclear translocation of P65. Furthermore, we found an important result, the Co-IP and IF colocalization results showed that LIPUS reduce the binding between YAP and RIPK1 compared to the IL-1β group (Fig. 7I, J), thereby regulating the NF-κB pathway and the progression of OA. Additionally, the IHC results revealed that compared with the DMM group, AAV9-YAP further increased the expression level of P-YAP, P-P65 and P-RIPK1 in the cartilage. However, there were fewer P-YAP, P-P65 and P-RIPK1 positive chondrocytes observed in the cartilage of DMM rats treated with LIPUS, indicating the inhibitory effect of LIPUS on the phosphorylation of YAP, P65 and RIPK1 (Fig. 7K, L). Together, these results demonstrated that LIPUS had significant therapeutic effects on inflammatory chondrocytes by regulating YAP and NF-κB signaling pathway.

### LIPUS protects rat against DMM-induced cartilage degeneration

**Fig. 8:**
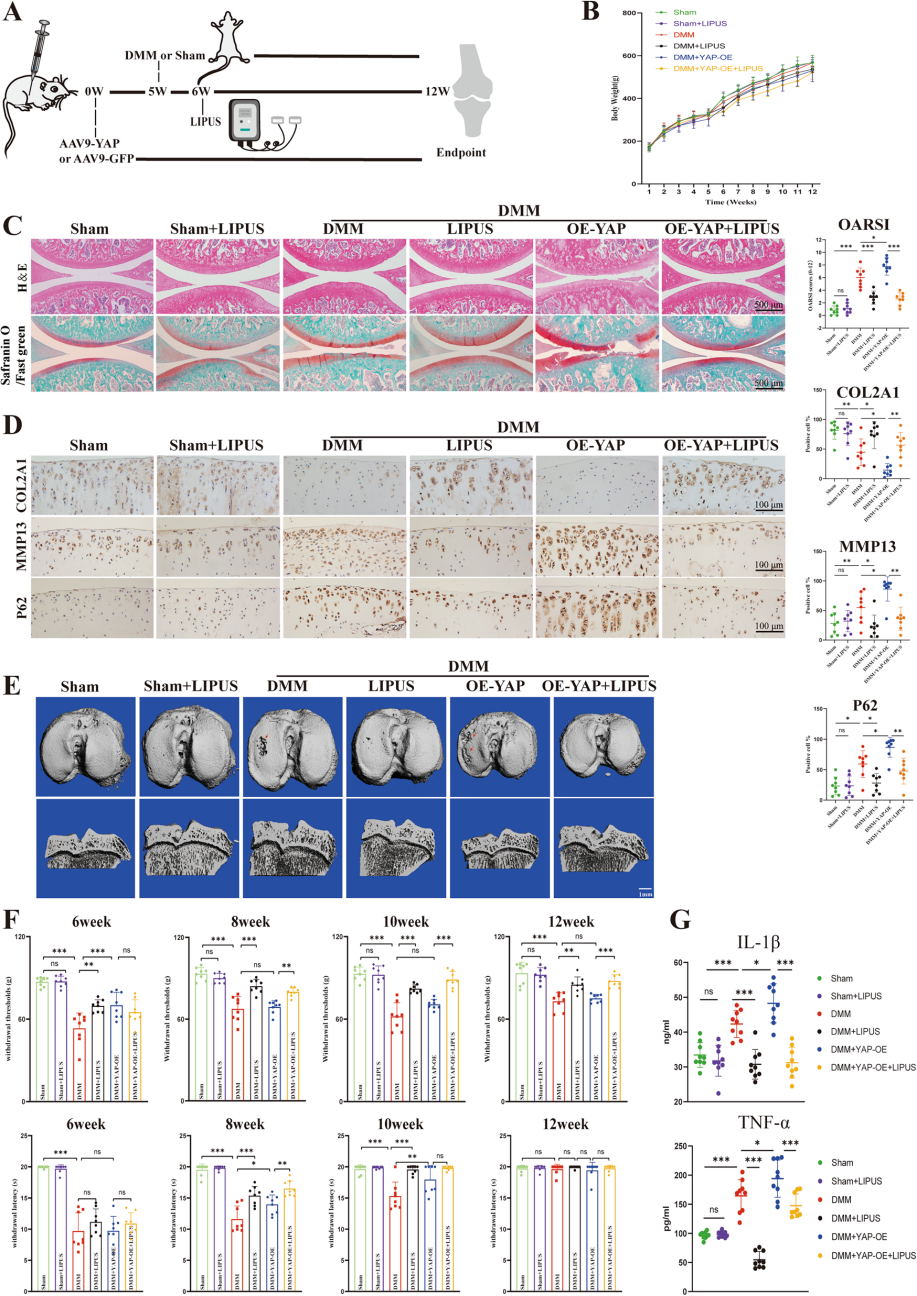
LIPUS inhibits cartilage degeneration in post-traumatic OA of rats. **A** Schematic diagram of animal experiment. **B** The body weight of rats. **C** H&E staining and Safranin O/Fast green of the six groups (scale bar: 500 μm). **D** Immunohistochemistry staining to show the COL2A1-positive cell, MMP13-positive cell and P62 -positive cell in cartilage of the six groups (scale bar: 100 μm). **E** The CT 3D reconstruction images of rats (scale bar: 1 mm). **F** Quantification of the estimation about mechanical and heat pain. **G** Serum concentration quantification of IL-1β and TNF-α. Data are shown as mean ± SD. *P < 0.05, **P < 0.01, ***P < 0.001

Considering the findings that LIPUS could relieve pain and promote cartilage repair, the effect of LIPUS on OA was investigated in vivo [17, 36]. Firstly, AAV9-YAP was intra-articular injected into rats to up-regulate the expression of YAP. Four weeks after injection, rats underwent either Sham or DMM surgery to establish the OA model. Subsequently, LIPUS was applied 1 week after surgery and continued for 6 weeks (Fig. 8A). The body weight among the groups showed no statistically significant difference (Fig. 8B). As expected, H&E and Safranin O/Fast green staining revealed that LIPUS administration significantly attenuated cartilage degeneration, as indicated by a lower OARSI score compared to the DMM group and the DMM + OE-YAP group (Fig. 8C). Consistent with above, the immunohistochemistry results showed that the highly expressed MMP13 and P62 in the cartilage of DMM rats were significantly aggravated in AAV-YAP-treated mice, which were markedly suppressed by LIPUS treatment. However, the expression of COL2A1exhibited the opposite trend. (Fig. 8D). Furthermore, the micro-CT image of the tibial plateau demonstrated that DMM surgery induced severe subchondral bone loss compared to Sham group, which was further exaggerated by YAP-AAV, but ameliorated by LIPUS treatment (Fig. 8E). Interestingly, the mechanical threshold of rats suffered DMM surgery decreased compared to the Sham group, but it was considerably increased after application of LIPUS. Notably, this therapeutic effect of LIPUS was observed only in the 8th and 10th week in the assessment of the nociception to heat (Fig. 8F), indicating that LIPUS effectively relieved pain in OA rats. Furthermore, we also examined the levels of inflammatory factors and found that DMM surgery and YAP overexpression increased the inflammatory factors (IL-1β and TNF-α) in the sera, while LIPUS reduced the level of inflammatory factors (Fig. 8G). In conclusion, these findings demonstrated that administration of LIPUS to the OA model attenuated cartilage degeneration, restore autophagy capacity and decrease inflammation in post-traumatic OA rats.

## Discussion

**Fig. 9:**
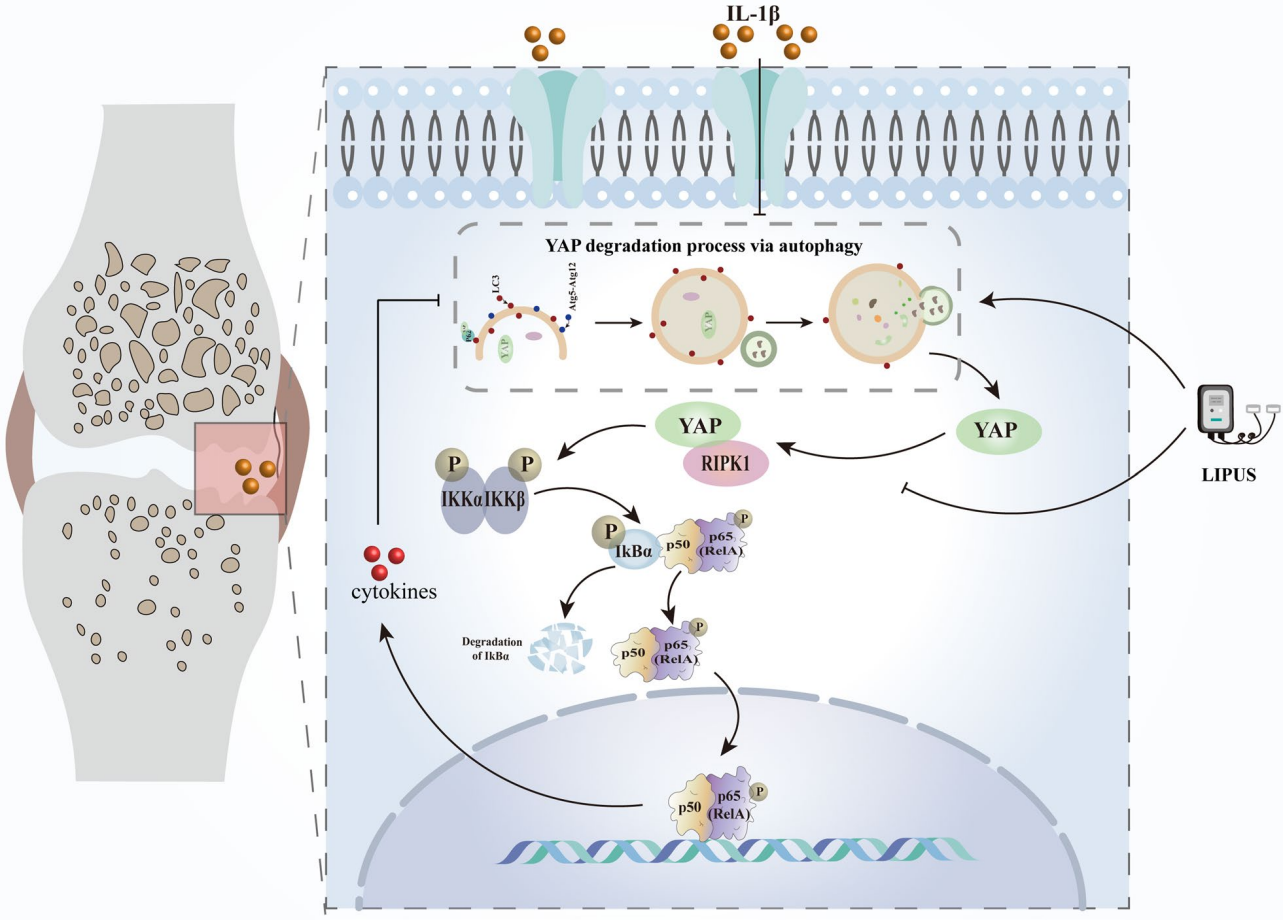
Schematic diagram of LIPUS inhibits the progression of OA by regulating the YAP–RIPK1–NF-κB axis

As reported, the Hippo/YAP signal transduction may be involved in regulating the physiological process of cartilage and is related to the pathogenesis of OA [37, 38]. However, the specific mechanism of its role in the occurrence and progression of OA is not yet clear. In this study, we found that the autophagy capacity of inflammatory chondrocytes was impaired, which leading to the accumulation of YAP and the expression of P-YAP in chondrocytes were increased. Subsequently, the increased intracellular YAP could bind and activate RIPK1, triggering the NF-κB signaling pathway and exacerbating chondrocyte inflammation and degradation of the ECM, ultimately leading to cartilage degradation. Interestingly, LIPUS suppressed the progression of OA by restoring autophagy capacity and reducing the binding between YAP and RIPK1. Therefore, enhancing autophagy capacity and inhibiting the YAP–RIPK1–NF-κB axis through LIPUS may provide a new therapeutic approach for OA (Fig. 9).

As reported, changes in autophagy-related genes that interact with the Hippo-YAP signaling pathway are essential for maintaining the physiological balance of the body [39, 40]. Meanwhile, excessive apoptosis of chondrocytes and a deficiency in protective autophagy capacity may be the pathogenesis of OA [41, 42]. Besides, when autophagy is relatively inactive, the activity of YAP increases [43]. Our study found that after treating chondrocytes with IL-1β, the autophagy capacity of chondrocytes declined while the expression of YAP increased. Additionally, we confirmed the interaction between P62 and YAP, indicating that YAP is directly related to autophagy in chondrocytes, and the accumulation of YAP is caused by impaired autophagy capacity.

Interestingly, the expression of YAP in OA shows different and even opposite changes in different studies [15, 44]. Considering the role of YAP in inflammatory-related diseases, elucidating of the mechanisms of YAP in osteoarthritis is important for more precisely targeting this molecule. Our results suggest that the expression of YAP increases in inflammatory chondrocytes and post-traumatic OA rats. Besides, IL-1β promoted the phosphorylation of YAP. To further confirm the connection between YAP and OA, the expression level of YAP was changed both in vivo and in vitro experiments. We observed that YAP overexpression accelerated the progression of OA, while knockdown of YAP expression reversed this change, indicating that YAP is closely connected to the occurrence and progression of OA. Nevertheless, the potential mechanism remains unclear. Deng et al. demonstrated that YAP block the activation of IKKα/β, thereby inhibiting the NF-κB signaling pathway [15]. In this study, we have confirmed for the first time that YAP can interact with RIPK1 to activate downstream NF-κB signaling pathways, thereby accelerating the progression of OA.

Recently, it has been found that LIPUS can mitigate the progression of some inflammatory diseases including OA. For example, Zhang et al. found that LIPUS improves synovial inflammation and painful gait patterns in DMM mice. Mechanistically, LIPUS up-regulates the level of macroautophagy/autophagy, accelerates the formation of SQSTM1-PKM complex and down-regulates the level of PKM2 in LPS-ATP-treated macrophages, thereby inhibiting IL-1β production [45]. Mitophagy is an important regulatory mode for cells to maintain normal function. A recent study showed that focused LIPUS can activate mitophagy, promote PGAM5 expression and dephosphorylation of FUNDC1 at Ser13, thereby improving the inflammatory response, anabolism and catabolism of OA chondrocytes induced by IL-1β [46]. In this study, we selected the optimal conditions for treating chondrocytes with LIPUS and found that when the intensity was 30 mW/cm^2^ and the duration was 20 min, LIPUS most significantly inhibited inflammation of chondrocytes. Furthermore, it also promoted ECM homeostasis and restored the decreased level of autophagy caused by IL-1β, which is consistent with the findings of previous studies. However, we selected the most effective conditions of intervention and focused on the new mechanisms behind, especially the transformation of mechanical mechanics. As reported, YAP is the substrate of autophagy, and mechanical signals can regulate the efficiency of autophagy by adjusting YAP signaling pathway, which promotes the fusion of autophagy vesicles and lysosomes [14, 40]. In addition, YAP is a mechanotransducer, which can sense the external mechanical stimulation to cells and convert it into cell-specific transcriptional program [47, 48]. Base on this background, we found that LIPUS inhibited the phosphorylation of YAP by regulating the effect of mechanical stress on cells. Furthermore, we also found that LIPUS reduced the interaction between YAP and RIPK1, thereby preventing the activation of the NF-κB signaling pathway, thus reducing the damage caused by the inflammatory mediators, restoring impaired autophagy capacity and promoting the degradation of YAP. As we all known, the present treatments including symptomatic treatment and joint replacement cannot reverse the progression of OA, it is necessary to research novel treatment methods, especially non-invasive treatment. Notably, more and more basic and clinical studies including our study confirmed the effectiveness of LIPUS in the treatment of osteoarthritis in the last decade, providing more evidence that LIPUS become a new option for the non-invasive treatment of OA [46, 49–52].

However, there are some limitations in this study. Firstly, although we have confirmed for the first time that YAP can bind to RIPK1, the specific binding region needs further elucidation, which will be the focus of our future research. Secondly, although many studies have confirmed the effectiveness of LIPUS, most of them are still in the stage of cell and animal experiments, which as same as our study [53]. There are few clinical experimental data on LIPUS in the treatment of OA and the intensity and effects are different [54, 55]. Therefore, it is important to note that there is no unified standard for the intensity and duration of LIPUS on the treatment of OA, and the specific regulatory parameters still need to be further optimized by preclinical studies, which is the future direction of us.

## Conclusions

In conclusion, this study investigated the specific mechanism of how YAP contributes to the occurrence and development of OA. Our findings demonstrate that the impaired autophagy leads to the accumulation of YAP, which in turn causes further injury of chondrocyte via the YAP–RIPK1–NF-κB axis. In addition, we have selected the most effective intensity and duration of the application of LIPUS and confirmed that LIPUS regulated the phosphorylation of YAP and reduced the binding of YAP and P-RIPK1, thereby significantly inhibiting IL-1β-induced chondrocyte inflammation and autophagy damage. Furthermore, we also demonstrated that LIPUS could significantly inhibit cartilage degeneration in post-traumatic OA rats. These findings further confirm the potential of LIPUS in the treatment of osteoarthritis and provide data on the selection of therapeutic parameters, which will provide new directions for the clinical application and scientific research of LIPUS.

## Supplementary Information

**Additional file 1: Figure S1.** The efficiency of knocking down or up-regulation the expression level of YAP through siRNA or plasmid. (A) qPCR of YAP relative expression level. (B, C) Western blot and quantitative analysis of the expression level of YAP. Data are shown as mean ± SD. *P < 0.05, **P < 0.01, ***P < 0.001.

## Data Availability

The datasets used and/or analyzed during the current study are available from the corresponding author [Tao Xu], on reasonable request.

## Abbreviations

ACAN: Aggrecan
ADAMTS5: A disintegrin and metalloproteinase with thrombospondin motifs 5
ATG5: Autophagy-related gene 5
AAV: Adeno-associated virus
ANOVA: Analysis of variance
COL2A1: Collagen type II α 1 chain
COX2: Cyclooxygenase-2
Co-IP: Co-immunoprecipitation
DMEM/F12: Dulbecco’s modified Eagle’s medium
DMM: Destabilization of medial meniscus
ECM: Extracellular matrix
ELISA: Enzyme-linked immunosorbent assay
H&E: Hematoxylin–eosin
IL-1β: Interleukin 1 beta
iNOS: Inducible nitric oxide synthase
IF: Immunofluorescence
LIPUS: Low-intensity pulsed ultrasound
LC3: Light chain 3β
LPS: Lipopolysaccharide
micro-CT: Micro-computed tomography
MMP3: Metalloproteinase 3
MMP13: Matrix metallopeptidase 13
NF-κB: Nuclear factor Kappa B
OA: Osteoarthritis
OARSI: Osteoarthritis Research Society International
PFA: Paraformaldehyde
PVDF: Polyvinylidene difluoride
Rapa: Rapamycin
SDS-PAGE: Sodium dodecyl sulfate-polyacrylamide gel electrophoresis
siRNA: Small interfering RNA
TBS: Tris-buffered saline
TBST: TBS Tween
SD: Sprague–Dawley
TNF-α: Tumor necrosis factor α
YAP: Yes-associated protein

## References

[1] Jang S, Lee K, Ju JH. Recent updates of diagnosis, pathophysiology, and treatment on osteoarthritis of the knee. Int J Mol Sci. 2021;22:2619. 33807695 10.3390/ijms22052619 PMC7961389

[2] Litwic A, Edwards MH, Dennison EM, Cooper C. Epidemiology and burden of osteoarthritis. Br Med Bull. 2013;105:185–99. 23337796 10.1093/bmb/lds038 PMC3690438

[3] Sharma L. Osteoarthritis of the knee. N Engl J Med. 2021;384:51–9. 33406330 10.1056/NEJMcp1903768

[4] Hunter DJ, Bierma-Zeinstra S. Osteoarthritis. Lancet. 2019;393:1745–59. 31034380 10.1016/S0140-6736(19)30417-9

[5] Jahr H, Brill N, Nebelung S. Detecting early stage osteoarthritis by optical coherence tomography? Biomarkers. 2015;20:590–6. 26862954 10.3109/1354750X.2015.1130190 PMC4819848

[6] Assi R, Quintiens J, Monteagudo S, Lories RJ. Innovation in targeted intra-articular therapies for osteoarthritis. Drugs. 2023;83:649–63. 37067759 10.1007/s40265-023-01863-y

[7] Levine B, Kroemer G. Autophagy in the pathogenesis of disease. Cell. 2008;132:27–42. 18191218 10.1016/j.cell.2007.12.018 PMC2696814

[8] Ballabio A, Bonifacino JS. Lysosomes as dynamic regulators of cell and organismal homeostasis. Nat Rev Mol Cell Biol. 2020;21:101–18. 31768005 10.1038/s41580-019-0185-4

[9] López de Figueroa P, Lotz MK, Blanco FJ, Caramés B. Autophagy activation and protection from mitochondrial dysfunction in human chondrocytes. Arthritis Rheumatol. 2015;67:966–76. 25605458 10.1002/art.39025 PMC4380780

[10] Caramés B, Hasegawa A, Taniguchi N, Miyaki S, Blanco FJ, Lotz M. Autophagy activation by rapamycin reduces severity of experimental osteoarthritis. Ann Rheum Dis. 2012;71:575–81. 22084394 10.1136/annrheumdis-2011-200557 PMC3294168

[11] Plouffe SW, Hong AW, Guan KL. Disease implications of the Hippo/YAP pathway. Trends Mol Med. 2015;21:212–22. 25702974 10.1016/j.molmed.2015.01.003 PMC4385444

[12] Misra JR, Irvine KD. The Hippo signaling network and its biological functions. Annu Rev Genet. 2018;52:65–87. 30183404 10.1146/annurev-genet-120417-031621 PMC6322405

[13] Zhu S, Wang X, Chen H, Zhu W, Li X, Cui R, Yi X, Chen X, Li H, Wang G. Hippo (YAP)-autophagy axis protects against hepatic ischemia–reperfusion injury through JNK signaling. Chin Med J. 2023. 10.1097/CM9.0000000000002727. 37232477 10.1097/CM9.0000000000002727 PMC10950187

[14] Lee YA, Noon LA, Akat KM, Ybanez MD, Lee TF, Berres ML, Fujiwara N, Goossens N, Chou HI, Parvin-Nejad FP, et al. Autophagy is a gatekeeper of hepatic differentiation and carcinogenesis by controlling the degradation of Yap. Nat Commun. 2018;9:4962. 30470740 10.1038/s41467-018-07338-z PMC6251897

[15] Deng Y, Lu J, Li W, Wu A, Zhang X, Tong W, Ho KK, Qin L, Song H, Mak KK. Reciprocal inhibition of YAP/TAZ and NF-κB regulates osteoarthritic cartilage degradation. Nat Commun. 2018;9:4564. 30385786 10.1038/s41467-018-07022-2 PMC6212432

[16] Harrison A, Alt V. Low-intensity pulsed ultrasound (LIPUS) for stimulation of bone healing—a narrative review. Injury. 2021;52(Suppl 2):S91–6. 34020780 10.1016/j.injury.2021.05.002

[17] Korstjens CM, van der Rijt RH, Albers GH, Semeins CM, Klein-Nulend J. Low-intensity pulsed ultrasound affects human articular chondrocytes in vitro. Med Biol Eng Comput. 2008;46:1263–70. 18853213 10.1007/s11517-008-0409-9

[18] Xia P, Ren S, Lin Q, Cheng K, Shen S, Gao M, Li X. Low-intensity pulsed ultrasound affects chondrocyte extracellular matrix production via an integrin-mediated p38 MAPK signaling pathway. Ultrasound Med Biol. 2015;41:1690–700. 25736607 10.1016/j.ultrasmedbio.2015.01.014

[19] Aimaijiang M, Liu Y, Zhang Z, Qin Q, Liu M, Abulikemu P, Liu L, Zhou Y. LIPUS as a potential strategy for periodontitis treatment: a review of the mechanisms. Front Bioeng Biotechnol. 2023;11:1018012. 36911184 10.3389/fbioe.2023.1018012 PMC9992218

[20] Li P, Zhang Z, Liu J, Xue H. LIPUS can promote osteogenesis of hPDLCs and inhibit the periodontal inflammatory response via TLR5. Oral Dis. 2023. 10.1111/odi.14807. 37983889 10.1111/odi.14807

[21] Hsu CH, Pan YJ, Zheng YT, Lo RY, Yang FY. Ultrasound reduces inflammation by modulating M1/M2 polarization of microglia through STAT1/STAT6/PPARγ signaling pathways. CNS Neurosci Ther. 2023;29:4113–23. 37401041 10.1111/cns.14333 PMC10651950

[22] Yang FY, Chan WH, Gao CY, Zheng YT, Ke CH. Transabdominal ultrasound alleviates LPS-induced neuroinflammation by modulation of TLR4/NF-κB signaling and tight junction protein expression. Life Sci. 2023;325: 121769. 37178865 10.1016/j.lfs.2023.121769

[23] Su WS, Wu CH, Song WS, Chen SF, Yang FY. Low-intensity pulsed ultrasound ameliorates glia-mediated inflammation and neuronal damage in experimental intracerebral hemorrhage conditions. J Transl Med. 2023;21:565. 37620888 10.1186/s12967-023-04377-z PMC10464049

[24] Cao Q, Liu L, Hu Y, Cao S, Tan T, Huang X, Deng Q, Chen J, Guo R, Zhou Q. Low-intensity pulsed ultrasound of different intensities differently affects myocardial ischemia/reperfusion injury by modulating cardiac oxidative stress and inflammatory reaction. Front Immunol. 2023;14:1248056. 37744362 10.3389/fimmu.2023.1248056 PMC10513435

[25] Wu CT, Yang TH, Chen MC, Chung YP, Guan SS, Long LH, Liu SH, Chen CM. Low intensity pulsed ultrasound prevents recurrent ischemic stroke in a cerebral ischemia/reperfusion injury mouse model via brain-derived neurotrophic factor induction. Int J Mol Sci. 2019;20:5169. 31635269 10.3390/ijms20205169 PMC6834125

[26] Xia P, Wang X, Wang Q, Wang X, Lin Q, Cheng K, Li X. Low-intensity pulsed ultrasound promotes autophagy-mediated migration of mesenchymal stem cells and cartilage repair. Cell Transplant. 2021;30:963689720986142. 33412895 10.1177/0963689720986142 PMC7797574

[27] Xia P, Wang Q, Song J, Wang X, Wang X, Lin Q, Cheng K, Chen A, Li X. Low-intensity pulsed ultrasound enhances the efficacy of bone marrow-derived mscs in osteoarthritis cartilage repair by regulating autophagy-mediated exosome release. Cartilage. 2022;13:19476035221093060. 35438034 10.1177/19476035221093060 PMC9137322

[28] Ahmadian E, Eftekhari A, Janas D, Vahedi P. Nanofiber scaffolds based on extracellular matrix for articular cartilage engineering: a perspective. Nanotheranostics. 2023;7:61–9. 36593799 10.7150/ntno.78611 PMC9760364

[29] Baran A, Fırat Baran M, Keskin C, Hatipoğlu A, Yavuz Ö, İrtegün Kandemir S, Adican MT, Khalilov R, Mammadova A, Ahmadian E, et al. Investigation of antimicrobial and cytotoxic properties and specification of silver nanoparticles (AgNPs) derived from *Cicer arietinum* L. green leaf extract. Front Bioeng Biotechnol. 2022;10: 855136. 35330628 10.3389/fbioe.2022.855136 PMC8940290

[30] Xie S, Li G, Hou Y, Yang M, Li F, Li J, Li D, Du Y. A synergistic bactericidal effect of low-frequency and low-intensity ultrasound combined with levofloxacin-loaded PLGA nanoparticles on *M. smegmatis* in macrophages. J Nanobiotechnol. 2020;18:107. 10.1186/s12951-020-00658-7 PMC7388535 32727616

[31] Khalilov R. Comprehensive review of advanced nano-biomaterials in regenerative medicine and drug delivery. Adv Biol Earth Sci. 2023;8(1):5–18.

[32] Jian Z, Li Y, Zhang C, Zhong W, Ai D, He Y, Song J. Low-intensity pulsed ultrasound attenuates periodontal ligament cells apoptosis by activating yes-associated protein-regulated autophagy. Ultrasound Med Biol. 2023;49:1227–37. 36878833 10.1016/j.ultrasmedbio.2023.01.008

[33] Puts R, Rikeit P, Ruschke K, Knaus P, Schreivogel S, Raum K. Functional regulation of YAP mechanosensitive transcriptional coactivator by focused low-intensity pulsed ultrasound (FLIPUS) enhances proliferation of murine mesenchymal precursors. PLoS ONE. 2018;13: e0206041. 30365513 10.1371/journal.pone.0206041 PMC6203358

[34] Sachs D, Cunha FQ, Poole S, Ferreira SH. Tumour necrosis factor-alpha, interleukin-1beta and interleukin-8 induce persistent mechanical nociceptor hypersensitivity. Pain. 2002;96:89–97. 11932065 10.1016/s0304-3959(01)00433-x

[35] Milanovic M, Kracht M, Schmitz ML. The cytokine-induced conformational switch of nuclear factor κB p65 is mediated by p65 phosphorylation. Biochem J. 2014;457:401–13. 24175631 10.1042/BJ20130780

[36] Rutjes AW, Nüesch E, Sterchi R, Jüni P. Therapeutic ultrasound for osteoarthritis of the knee or hip. Cochrane Database Syst Rev. 2010. 10.1002/14651858.CD003132.pub2. 20091539 10.1002/14651858.CD003132.pub2

[37] Sun K, Guo J, Guo Z, Hou L, Liu H, Hou Y, He J, Guo F, Ye Y. The roles of the Hippo-YAP signalling pathway in cartilage and osteoarthritis. Ageing Res Rev. 2023;90: 102015. 37454824 10.1016/j.arr.2023.102015

[38] Gong Y, Li SJ, Liu R, Zhan JF, Tan C, Fang YF, Chen Y, Yu B. Inhibition of YAP with siRNA prevents cartilage degradation and ameliorates osteoarthritis development. J Mol Med. 2019;97:103–14. 30465058 10.1007/s00109-018-1705-y

[39] Wang D, He J, Huang B, Liu S, Zhu H, Xu T. Emerging role of the Hippo pathway in autophagy. Cell Death Dis. 2020;11:880. 33082313 10.1038/s41419-020-03069-6 PMC7576599

[40] Totaro A, Zhuang Q, Panciera T, Battilana G, Azzolin L, Brumana G, Gandin A, Brusatin G, Cordenonsi M, Piccolo S. Cell phenotypic plasticity requires autophagic flux driven by YAP/TAZ mechanotransduction. Proc Natl Acad Sci USA. 2019;116:17848–57. 31416916 10.1073/pnas.1908228116 PMC6731754

[41] Shi S, Tian T, Li Y, Xiao D, Zhang T, Gong P, Lin Y. Tetrahedral framework nucleic acid inhibits chondrocyte apoptosis and oxidative stress through activation of autophagy. ACS Appl Mater Interfaces. 2020;12:56782–91. 33289541 10.1021/acsami.0c17307

[42] Yang H, Wen Y, Zhang M, Liu Q, Zhang H, Zhang J, Lu L, Ye T, Bai X, Xiao G, Wang M. MTORC1 coordinates the autophagy and apoptosis signaling in articular chondrocytes in osteoarthritic temporomandibular joint. Autophagy. 2020;16:271–88. 31007149 10.1080/15548627.2019.1606647 PMC6984599

[43] Mikula M, Najjar S, El Jabbour T, Dalvi S, Umrau K, Li H, Koo BH, Lee H. Increased cytoplasmic yes-associated protein (YAP) expression in mismatch repair protein-proficient colorectal cancer with high-grade tumor budding and reduced autophagy activity. Appl Immunohistochem Mol Morphol. 2021;29:305–12. 33264106 10.1097/PAI.0000000000000888

[44] Zhang Q, Fang X, Zhao W, Liang Q. The transcriptional coactivator YAP1 is overexpressed in osteoarthritis and promotes its progression by interacting with Beclin-1. Gene. 2019;689:210–9. 30496783 10.1016/j.gene.2018.11.068

[45] Zhang B, Chen H, Ouyang J, Xie Y, Chen L, Tan Q, Du X, Su N, Ni Z, Chen L. SQSTM1-dependent autophagic degradation of PKM2 inhibits the production of mature IL1B/IL-1β and contributes to LIPUS-mediated anti-inflammatory effect. Autophagy. 2020;16:1262–78. 31500508 10.1080/15548627.2019.1664705 PMC7469634

[46] Ye H, Li D, Wei X, Yu L, Jia L. Focused low-intensity pulsed ultrasound alleviates osteoarthritis via restoring impaired FUNDC1-mediated mitophagy. iScience. 2023;26: 107772. 37720103 10.1016/j.isci.2023.107772 PMC10504546

[47] Mannion AJ, Zhao H, Zhang Y, von Wright Y, Bergman O, Roy J, Saharinen P, Holmgren L. Regulation of YAP promotor accessibility in endothelial mechanotransduction. Arterioscler Thromb Vasc Biol. 2024;44(3):666–89. 38299356 10.1161/ATVBAHA.123.320300 PMC10880945

[48] Panciera T, Azzolin L, Cordenonsi M, Piccolo S. Mechanobiology of YAP and TAZ in physiology and disease. Nat Rev Mol Cell Biol. 2017;18:758–70. 28951564 10.1038/nrm.2017.87 PMC6192510

[49] Zhou XY, Zhang XX, Yu GY, Zhang ZC, Wang F, Yang YL, Li M, Wei XZ. Effects of low-intensity pulsed ultrasound on knee osteoarthritis: a meta-analysis of randomized clinical trials. Biomed Res Int. 2018;2018:7469197. 30105243 10.1155/2018/7469197 PMC6076961

[50] Gurkan I, Ranganathan A, Yang X, Horton WE Jr, Todman M, Huckle J, Pleshko N, Spencer RG. Modification of osteoarthritis in the guinea pig with pulsed low-intensity ultrasound treatment. Osteoarthr Cartil. 2010;18:724–33. 10.1016/j.joca.2010.01.006 PMC2873836 20175971

[51] Jia L, Li D, Wei X, Chen J, Zuo D, Chen W. Efficacy and safety of focused low-intensity pulsed ultrasound versus pulsed shortwave diathermy on knee osteoarthritis: a randomized comparative trial. Sci Rep. 2022;12:12792. 35896688 10.1038/s41598-022-17291-z PMC9329394

[52] Uddin SMZ, Komatsu DE. Therapeutic potential low-intensity pulsed ultrasound for osteoarthritis: pre-clinical and clinical perspectives. Ultrasound Med Biol. 2020;46:909–20. 31959508 10.1016/j.ultrasmedbio.2019.12.007

[53] Lee W, Georgas E, Komatsu DE, Qin YX. Daily low-intensity pulsed ultrasound stimulation mitigates joint degradation and pain in a post-traumatic osteoarthritis rat model. J Orthop Transl. 2024;44:9–18. 10.1016/j.jot.2023.09.002 PMC10753057 38161708

[54] Chen H, Wang Z, Zhang X, Sun M. Effects of low-intensity pulsed ultrasound on knee osteoarthritis: a systematic review and meta-analysis of randomized controlled trials. Clin Rehabil. 2022;36:1153–69. 35535403 10.1177/02692155221097035 PMC9354068

[55] Jo NG, Ko MH, Won YH, Park SH, Seo JH, Kim GW. The efficacy of low-intensity pulsed ultrasound on articular cartilage and clinical evaluations in patients with knee osteoarthritis. J Back Musculoskelet Rehabil. 2022;35:1381–9. 35754261 10.3233/BMR-210357

