# Correction to: Low-intensity pulsed ultrasound delays the progression of osteoarthritis by regulating the YAP–RIPK1–NF-κB axis and influencing autophagy

**DOI:** 10.1186/s12967-026-07815-w

**Published:** 2026-02-13

**Authors:** Chunran Pan, Fan Lu, Xiaoxia Hao, Xiaofeng Deng, Jiawei Liu, Kai Sun, Wenjie Hou, Xingru Shang, Ruimin Chi, Fengjing Guo, Tao Xu

**Affiliations:** 1 Department of Rehabilitation, Tongji Hospital, Tongji Medical College, Huazhong University of Science and Technology, Wuhan, China; 2 Department of Orthopedics, Tongji Hospital, Tongji Medical College, Huazhong University of Science and Technology, Wuhan, China

**Correction to: J Transl Med 22, 286 (2024)**

**https://doi.org/10.1186/s12967-024-05086-x**

Following publication of the original article [1], the authors reported that the first image on the left of Fig. 8Ewas inadvertently reused in the second image on the left of Fig. 8E.

The incorrect version of Fig. 8 was:

**Fig. 8:**
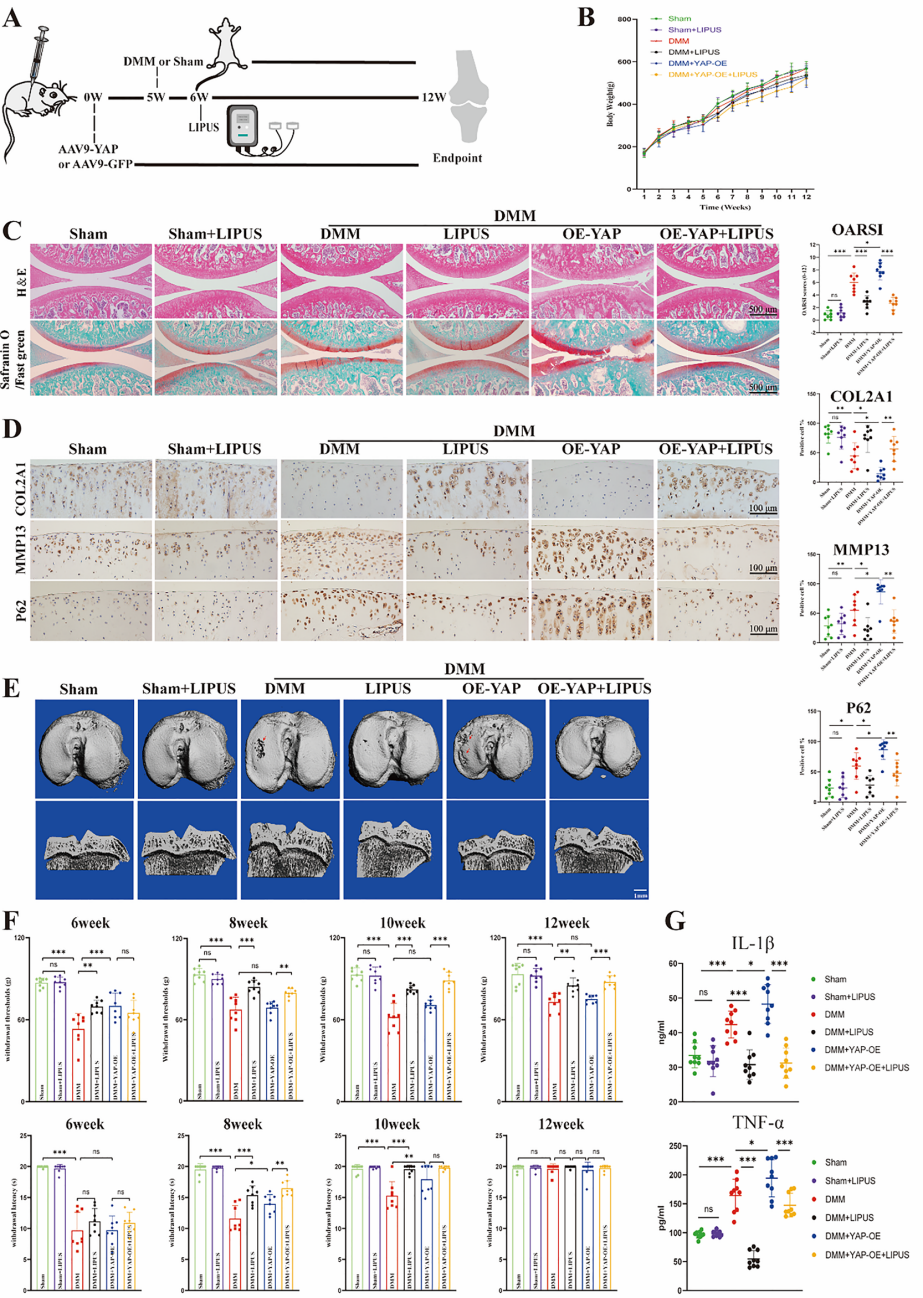
LIPUS inhibits cartilage degeneration in post-traumatic OA of rats. **A** schematic diagram of animal experiment. **B** the body weight of rats. **C** H&E staining and safranin O/Fast green of the six groups (scale bar: 500 μm). **D** immunohistochemistry staining to show the COL2A1-positive cell, MMP13-positive cell and P62 -positive cell in cartilage of the six groups (scale bar: 100 μm). **E** the CT 3D reconstruction images of rats (scale bar: 1 mm). **F** quantification of the estimation about Mechanical and heat pain. **G** serum concentration quantification of IL-1β and TNF-α. Data are shown as mean ± SD. *p < 0.05, **p < 0.01, ***p < 0.001

The correct Fig. 8 is:

**Fig. 8:**
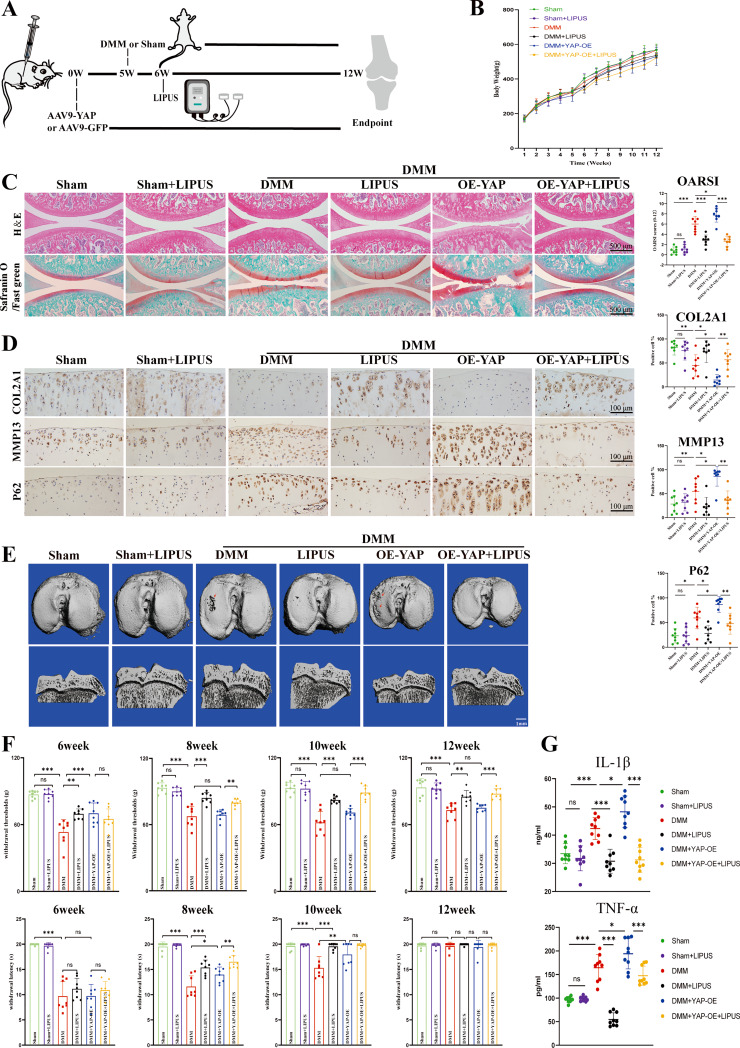
LIPUS inhibits cartilage degeneration in post-traumatic OA of rats. **A** schematic diagram of animal experiment. **B** the body weight of rats. **C** H&E staining and safranin O/Fast green of the six groups (scale bar: 500 μm). **D** immunohistochemistry staining to show the COL2A1-positive cell, MMP13-positive cell and P62 -positive cell in cartilage of the six groups (scale bar: 100 μm). **E** the CT 3D reconstruction images of rats (scale bar: 1 mm). **F** quantification of the estimation about Mechanical and heat pain. **G** serum concentration quantification of IL-1β and TNF-α. Data are shown as mean ± SD. *p < 0.05, **p < 0.01, ***p < 0.001

The original article [1] has been updated.
