# Supplementary figures and images for: Low-intensity pulsed ultrasound delays the progression of osteoarthritis by regulating the YAP–RIPK1–NF-κB axis and influencing autophagy
Source: J Transl Med. 2024 Mar 16;22:286. doi: 10.1186/s12967-024-05086-x (PMC10943805; doi:10.1186/s12967-024-05086-x)

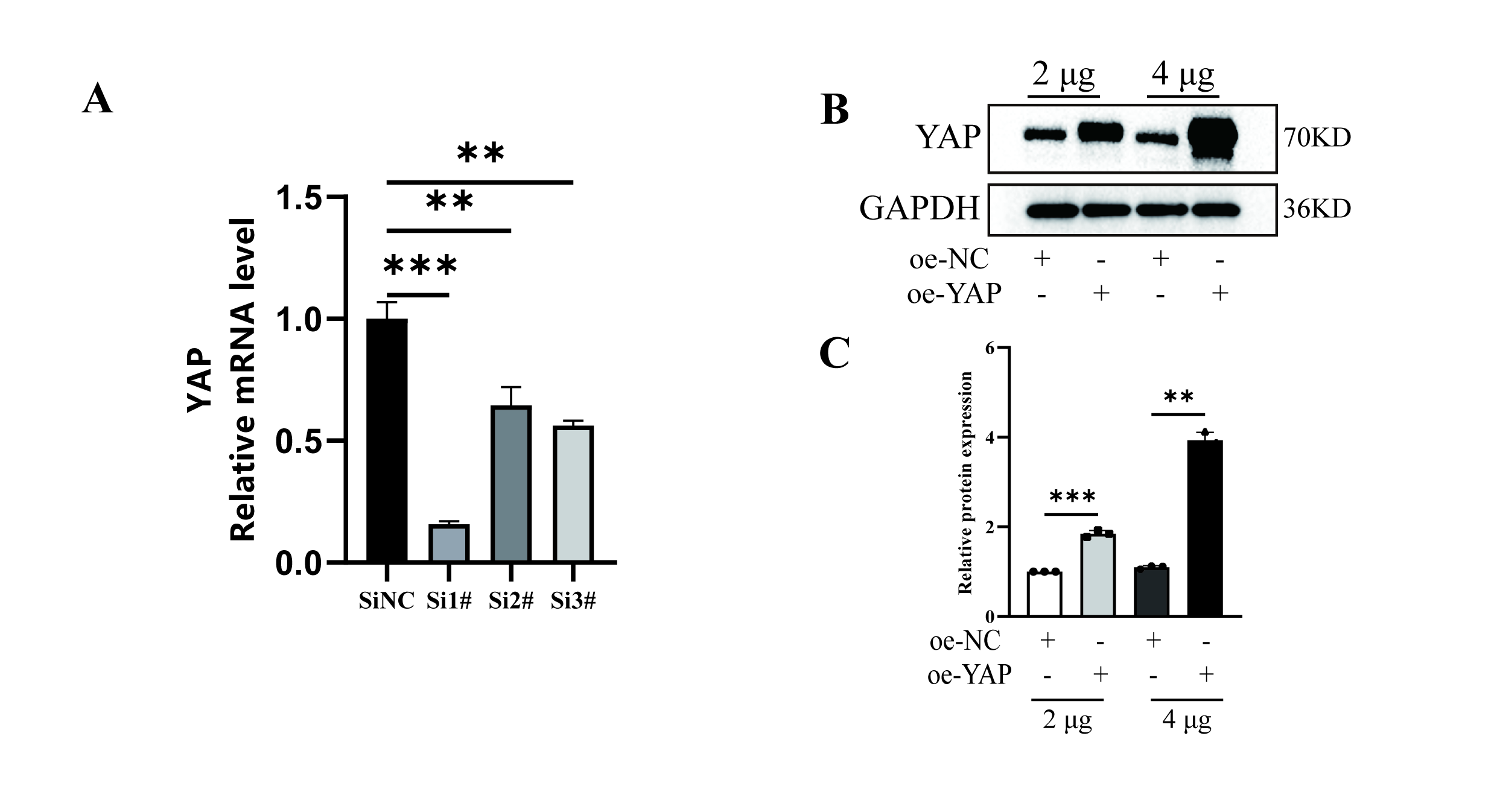

Supplement: Supplementary file 1 — Additional file 1: Figure S1. The efficiency of knocking down or up-regulation the expression level of YAP through siRNA or plasmid. (A) qPCR of YAP relative expression level. (B, C) Western blot and quantitative analysis of the expression level of YAP. Data are shown as mean ± SD. *P < 0.05, **P < 0.01, ***P < 0.001. [file 12967_2024_5086_MOESM1_ESM.tif]
